# Discovery of pyrano[2,3-*d*]pyrimidine-2,4-dione derivatives as novel PARP-1 inhibitors: design, synthesis and antitumor activity†

**DOI:** 10.1039/d0ra10321g

**Published:** 2021-01-22

**Authors:** Nour E. A. Abd El-sattar, Eman H. K. Badawy, Eman Z. Elrazaz, Nasser S. M. Ismail

**Affiliations:** Department of Chemistry, Organic Labs, Computational Chemistry Lab, Faculty of Science, Ain Shams University Cairo 11566 Egypt nourel-dinahmed@sci.asu.edu.eg; Department of Pharmaceutical Chemistry, Faculty of Pharmacy, Ain Shams University Abbassia 11566 Cairo Egypt eman_elrazaz@pharma.asu.edu.eg; Pharmaceutical Chemistry Department, Faculty of Pharmaceutical Sciences and Pharmaceutical Industries, Future University in Egypt (FUE) Cairo 12311 Egypt

## Abstract

Poly(ADP-ribose) polymerases-1 (PARP-1) are involved in DNA repair damage and so PARP-1 inhibitors have been used as potentiators in combination with DNA damaging cytotoxic agents to compromise the cancer cell DNA repair mechanism, resulting in genomic dysfunction and cell death. In this study, we report the synthesis of a novel series of pyrano[2,3-*d*]pyrimidine-2,4-dione analogues as potential inhibitors against PARP-1. All the newly synthesized compounds were evaluated for their inhibitory activity towards PARP-1 and examined for their anti-proliferative activity against MCF-7 and HCT116 human cancer cell lines. The synthesized compounds showed promising activity where compounds S2 and S7 emerged as the most potent PARP-1 inhibitors with an IC_50_ value of 4.06 ± 0.18 and 3.61 ± 0.15 nM, respectively compared to that of Olaparib 5.77 nM and high cytotoxicity against MCF-7 with IC_50_ 2.65 ± 0.05 and 1.28 ± 1.12 μM, respectively (Staurosporine 7.258 μM). Compound S8 remarkably showed the highest cell growth inhibition against MCF-7 and HCT116 with an IC_50_ value of 0.66 ± 0.05 and 2.76 ± 0.06 μM, respectively. Furthermore, molecular docking of the compounds into the PARP-1 active site was performed to explore the probable binding mode. Finally, most of the synthesized compounds were predicted to have good pharmacokinetics properties in a theoretical kinetic study.

## Abstract 2

PARP-1 are involved in DNA repair damage and so PARP-1 inhibitors have been used as potentiators in combination with DNA damaging cytotoxic agents to compromise the cancer cell DNA repair mechanism, resulting in genomic dysfunction and cell death.

## Graphical abstract

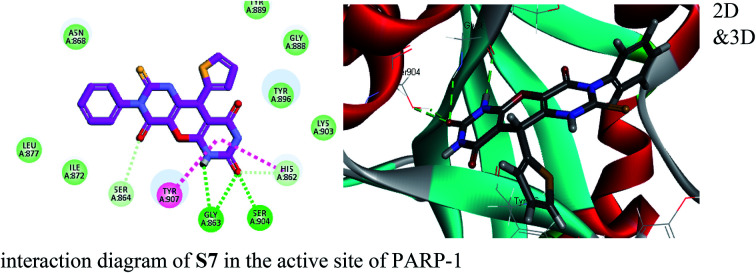

## 1 Introduction

PARP-1 has received great attention as a promising anti-cancer therapeutic target.^1^ PARP-1 is a highly conserved DNA-binding protein and is the most extensively expressed member of the poly(ADP-ribose) polymerases (PARPs) family which is composed of 18 members. They regulate a number of cellular processes including surveillance of genome integrity, cellular differentiation, regulation of gene transcription, inflammation, mitosis, cell cycle progression, initiation of DNA damage response and apoptosis.^2^ PARP-1 is a known sensor of DNA damage as it is responsible for DNA base excision repair (BER) and DNA single-strand break (SSB) repair.^3^ Damaged DNA activates PARP-1 to cleave its substrate nicotinamide adenine dinucleotide (NAD+) and to catalyze the addition of ADP-ribose units to it and to nuclear target proteins to recruit BER components to facilitate DNA repair process and cell survival.^4–7^ Increased PARP-1 expression is sometimes observed in melanomas, breast cancer, lung cancer, and other neoplastic diseases.^8^

It has been disclosed that in BRCA1/2-mutant cancer cells, inhibition of PARP1 is synthetically lethal due to their dependence on PARP-1 activity for DNA (base excision) repair and subsequently survival.^9^ So PARP-1 inhibitors have shown success when used as monotherapy for treating genetically DNA repair-defective cancers. PARP-1 inhibitors have not only been used in BRCA1/2 deficient cancers but also used in combination therapy with DNA-damaging therapeutics to improve their potencies by blocking DNA-repairing process.

**Fig. 1:**
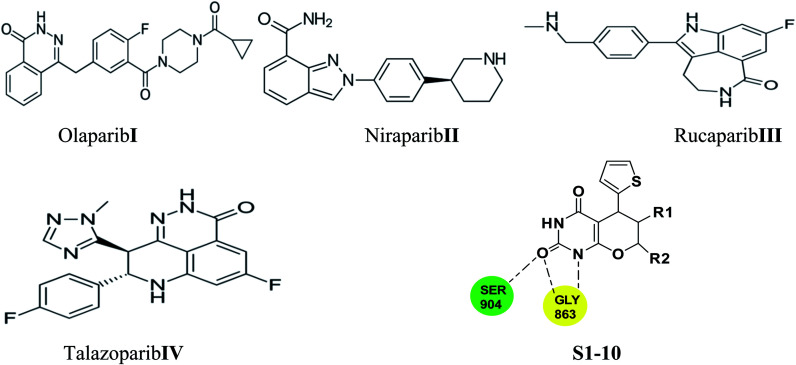
Approved PARP-1 inhibitors (Compounds I–IV) and our proposal compounds S1–10.

Few PARP-1 inhibitors have been discovered and FDA approved *e.g.*, Olaparib I,^10^ Niraparib II,^11^ Rucaparib III^12^ and Talazoparib IV (Fig. 1).^13^

The catalytic pocket of PARP-1 was usually characterized as two sub-pockets, one of the binding sites is occupied by the nicotinamide-ribose (NI site) and the other is the adenine-ribose binding site (AD site). Most of the reported PARP-1 inhibitors can bind the NI site through hydrogen bonds with residues Ser904 and Gly863, and additional pi–pi stacking interaction with Tyr907. Compared with the NI site, the AD site is large enough to accommodate diverse structure motifs.^14–17^

Herein, we employed pyrano[2,3-*d*] pyrimidine 2,4 dione analogues as a core structure to occupy the NI site and to interact with Ser904 and Gly863 through hydrogen bonds in an attempt to discover novel PARP-1 inhibitors.

## 2. Result and discussion

### 2.1. Chemistry

**Scheme 1:**
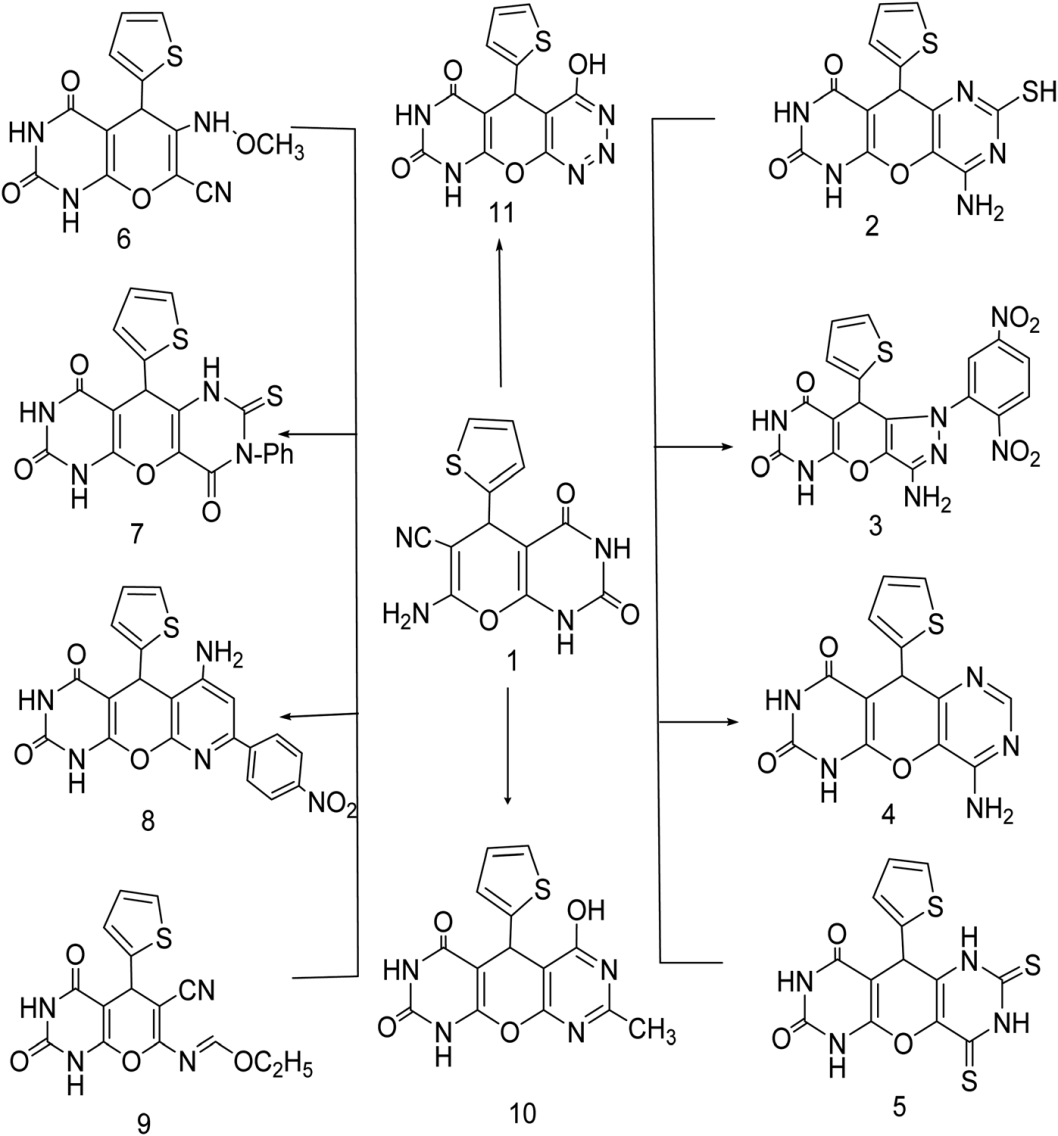
Synthesis of a novel series of pyrano[2,3-*d*] pyrimidine 2,4 dione (1–11).

Green synthesis of 7-amino-2,4-dioxo-5-(thiophen-2-yl)-2,3,4,5-tetrahydro-1*H*-pyrano[2,3-*d*]pyrimidine-6-carbonitrile (1) by treating cyclic compounds containing active methylene group with thiophen-2-carbaldehyde and malononitrile in solution of water : ethanol (1 : 1 ratio) as three component system for 2 h. The structure of the obtained products was confirmed using elemental analysis, spectroscopic data as well as chemical reaction (Scheme 1).^18–23^

Compound (1) was fused with thiourea then dissolved in a solution of sodium hydroxide to produce the corresponding 4-amino-2-mercapto-10-(thiophen-2-yl)-6*H*-pyrano[2,3-*d*:5,6-*d*']dipyrimidine-7,9(8*H*,10*H*)dione (2). In compound 2 stretching vibration was found in IR analysis of the (NH_2_) and 2(NH) groups at *ν* 3420.87, 3281.06, 3256.68 and 3163.37 cm^−1^. This IR pattern appears to be consistent with the assigned structure.

New pyrazolo derivatives were prepared by reacting 7-amino-2,4-dioxo-5-(thiophen-2-yl)-2,3,4,5-tetrahydro-1*H*-pyrano[2,3-*d*]pyrimidine-6-carbonitrile (1) with 2,4 dinitrophenyl hydrazine in 1,4-dioxane forming the corresponding new derivative 3-amino-1-(2,5-dinitrophenyl)-9-(thiophen-2-yl)-7,9-dihydropyrazolo[3′,4':5,6]pyrano[2,3-*d*] pyrimidine-6,8(1*H*,5*H*)-dione (3). The structure of compound (3) was characterized using spectroscopic and elemental analysis. IR spectroscopy of compound (3) showed the presence of two NH_2_ bands at 3420.17, 3365.15 cm^−1^, two NH bands at 3256.68 and 3163.37 cm^−1^ and two carbonyl bands at 1752.22 and 1694.82 cm^−1^ indicating the presence of unreacted cyano group. Furthermore, ^1^H-NMR spectra verified the structure by the appearance of three exchangeable signals at 9.12, 10.57 ppm for two NH and signals at 9.42–9.46 ppm for NH_2_.

The transformation of (1) into pyranodipyrimidine derivatives was achieved by its reaction with formamide and carbon disulfide respectively, under reflux. The structure of compound (4) was confirmed by their IR spectra through disappearance of CN with the appearance of two bands at 3214.28, 3111.74 cm^−1^ assignable to NH and NH_2_ group at 3470.68, 3249.52 cm^−1^, are good evidence for the structure given to this compound. While IR spectrum of compound (5) revealed bands at 3470.68, 3249.52, 3214.28 and 3111.74 cm^−1^ characteristic for 4NH groups, respectively, while it's ^1^H-NMR spectrum showed an exchangeable singlet signal of the NH proton at *δ* 7.34, 11.236, 11.277 ppm. Moreover, refluxing compound 1 with acetic anhydride, afforded the 6-(methoxyamino)-2,4-dioxo-5-(thiophen-2-yl)-2,3,4,5-tetrahydro-1*H*-pyrano[2,3-*d*]pyrimidine-7-carbonitrile (6). IR spectroscopy of (6) showed the presence of three NH bands at 3468.7, 3254.05 and 3196.21 cm^−1^, two carbonyl bands at 1730.52, 1690.42 cm^−1^ and one CN band at 2074.78 cm^−1^, indicating open chain structure. Furthermore, ^1^H-NMR spectra verified the structure by the appearance of three exchangeable NH signals at 2.47, 11.23 and 11.27 ppm. Compound (1) was subjected to reaction with phenyl isothiocyanate in dry pyridine to furnish the corresponding 3-phenyl-10-(thiophen-2-yl)-2-thioxo-2,3-dihydro-1*H*-pyrano[2,3-*d*:5,6-*d*']dipyrimidine-4,7,9(6*H*,8*H*,10*H*)-trione (7). It has been observed in compound (7) the absence of the cyanide peak in their IR spectra and the appearance of the imino group of pyrimidine moiety which proves the formation of cyclic compound (Scheme 1) (see Experimental).

Also cyclization of compound (1) with *p*-nitroacetophenone in the presence of triethylamine as catalyst yielded 6-amino-8-(4-nitrophenyl)-5-(thiophen-2-yl)-5,5*a*-dihydro-1*H*-pyrido[3′,2':5,6]pyrano[2,3-*d*]pyrimidine-2,4(3*H*,9*aH*)-dione (8). The formation of compound (8) was confirmed by the appearance of a signal at 6.95, 7.72 (s, 1H, 2NH, D_2_O exchangeable), 7.98 (s, 1H, NH_2_, D_2_O exchangeable), 8.18–8.36 (d, 4H, ArH) in ^1^H-NMR spectra, respectively, also reaction of compound (1) with triethylorthoformate, yielded (*E*)-ethyl *N*-(6-cyano-2,4-dioxo-5-(thiophen-2-yl)-2,3,4,5-tetrahydro-1*H*-pyrano[2,3-*d*]pyrimidin-7-yl)formimidate (9). In the ^1^H-NMR spectra of (9) a singlet peak at 8.71, 11.23 ppm (s, 1H, 2NH, D_2_O exchangeable) were found. Furthermore, in the IR spectra, the bands at 2069.45 cm^−1^ (CN) and two bands at 3199.9, 3147.11 cm^−1^ (NH).

New pyrimidine derivatives were prepared by treatment of compound (1) with a mixture of hydrochloric acid and acetic acid (1 : 3) giving 6-hydroxy-8-methyl-5-(thiophen-2-yl)-1*H*-pyrano[2,3-*d*:6,5-*d*'] dipyrimidine-2,4(3*H*,5*H*)-dione (10). ^1^H-NMR showed two exchangeable signals at 11.23, 11.27 ppm referred to NH and OH. IR spectroscopy provided two NH bands at 3125.16, 3040.10 cm^−1^ and one OH bands at 3450 cm^−1^. Moreover, two carbonyl bands appeared.

Diazotization of compound (1) with sodium nitrite and conc. Hydrochloric acid in the presence of acetic acid led to the formation of 4-hydroxy-5-(thiophen-2-yl)-9,10*a*-dihydro-4*aH*-pyrimido[5′,4':5,6]pyrano[2,3-*d*][1,2,3]triazine-6,8(5*H*,7*H*)-dione (11). The structure was confirmed by infrared spectrum which revealed no absorption in the CN region; furthermore, it displayed absorption bands at 3199.19 and 3442.41 cm^−1^ as a broad band of NH, OH.

### 2.2. Biological evaluation

The biological evaluation was accomplished through testing both enzyme inhibition activity and anti-proliferative activity. The enzymatic activity of the synthesized compounds was assessed against PARP-1.

#### 2.2.1 *In vitro* PARP-1 inhibitory assay

The synthesized compounds were evaluated for their PARP-1 inhibitory activity. As shown in Table 1, most of the synthesized compounds displayed excellent inhibitory activities against PARP-1 with IC_50_ values ranging from 3.61 nM to 114 nM compared to the reference drug Olaparib. Compounds S2 and S7 showed a higher potency than Olaparib. S4, S5, S8 and S10 showed a slightly less potency then Olaparib. Compounds S1, S6 and S9 showed the least potencies. The obtained result suggesting that the presence of a heterocycle fused with the pyrano[2,3-*d*]pyrimidine 2,4 dione enhances the potency.

**Table 1:** *In vitro* inhibitory activity of the synthesized compounds against PARP-1

Compound	IC_50_ (nM)	pIC_50_
S1	49.06 ± 2.32	7.31
S2	4.06 ± 0.18	8.39
S3	71.41 ± 4.12	7.15
S4	14.94 ± 1.03	7.82
S5	11.07 ± 0.76	7.96
S6	114.95 ± 7.22	6.94
S7	3.61 ± 0.15	8.44
S8	15.79 ± 0.86	7.80
S9	48.55 ± 3.25	7.31
S10	16.16 ± 0.88	7.79
Olaparib	5.77 ± 0.26	8.24

#### 2.2.2 Cell proliferation inhibition of synthesized compounds

All the synthesized compounds were further evaluated for their inhibition of cell proliferation with MCF-7 and HCT116 human cancer cell lines. All the synthesized compounds displayed moderate to good inhibition against MCF7 cells compared to the reference drug Staurosporine with IC_50_ ranging from 0.66 ± 0.05 to 12.68 ± 0.12 μM. Also, the compounds showed good inhibition against HCT116 cells ranging from 2.76 ± 0.06 to 46.86 ± 1.82 μM (Table 2).

**Table 2:** Antitumor activity against MCF-7 and HCT116 cells

Sample code	Results	
	IC_50_ (μM)	
	MCF-7	HCT116
S1	12.68 ± 0.12	24.07 ± 1.14
S2	2.65 ± 0.05	9.31 ± 0.34
S3	3.17 ± 1.04	2.80 ± 0.07
S4	0.87 ± 0.07	6.38 ± 0.11
S5	4.95 ± 0.07	7.41 ± 0.18
S6	3.12 ± 0.11	2.85 ± 0.03
S7	1.28 ± 1.12	46.86 ± 1.82
S8	0.66 ± 0.05	2.76 ± 0.06
S9	8.77 ± 0.15	2.90 ± 0.04
S10	4.48 ± 0.43	12.14 ± 0.64
Staurosporine	7.25 ± 0.13	6.94 ± 0.21

## 3. *In silico* studies

### 3.1. Molecular docking study

**Fig. 2:**
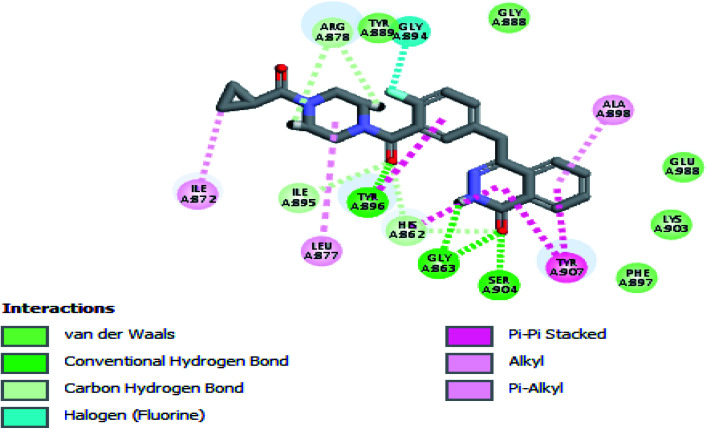
2D interaction diagram showing Olaparib (lead compound) docking pose interactions with the key amino acids in the PARP-1 active site.

The crystal structure of the ligand Olaparib complexed with PARP-1 (PDB ID: 5DS3) is shown in Fig. 2. In order to explore the binding mode of the designed pyrano[2,3-*d*]pyrimidine 2,4 dione derivatives, we obtained and analyzed the docked structures of the synthesized compounds within the catalytic site of PARP-1 and compared it with the that of Olaparib.

**Fig. 3:**
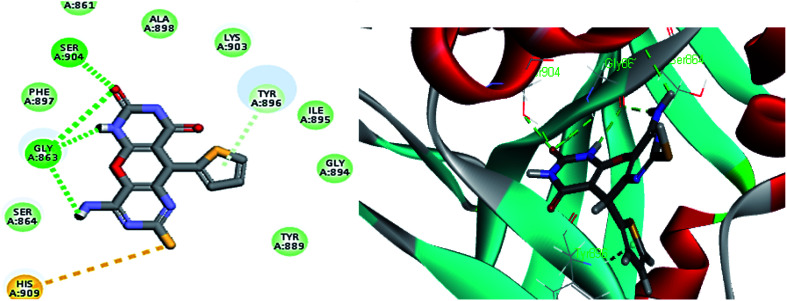
2D and 3D interaction diagram of S2 in the active site of PARP-1.

**Fig. 4:**
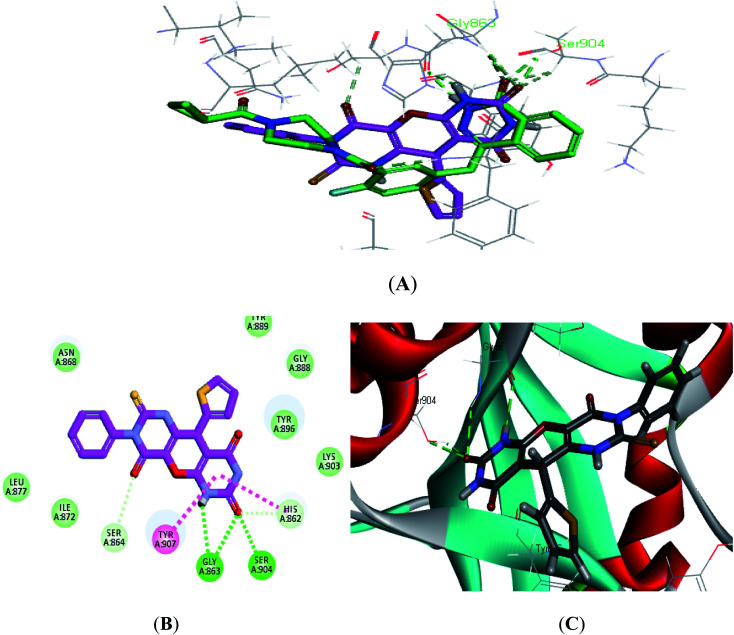
(A) Alignment of S7 (purple) with Olaparib (green), (B) 2D interaction diagram of S7 in the active site of PARP-1 and (C) 3D interaction diagram of S7 in the active site of PARP-1.

**Fig. 5:**
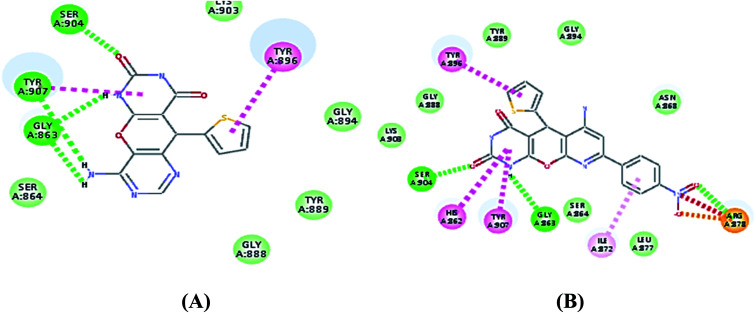
(A) 2D interaction diagram of S4 in the active site of PARP-1 and (B) 2D interaction diagram of S8 in the active site of PARP-1.

As expected, the pyrano[2,3-*d*] pyrimidine 2,4 dione scaffold occupied the NI-site and interacted with Ser904 through a hydrogen bond with the carbonyl group of the ring, also all the compounds interacted with Gly863 through two characteristic hydrogen bonds with the carbonyl group and NH of the ring. Also all compounds showed π–π stacking interactions with Tyr907 and His862. All these interactions were present in the reference Olaparib (Fig. 2–5).

As shown in Table 3 the docking results were consistent with the PARP-1 enzyme assay where the potent compounds showed good affinity to the enzyme as illustrated with their docking score. Interestingly, compound S2, which had an IC_50_ of 4.06 nM, the pyrimidine ring showed two additional interactions, the amino group did a hydrogen bond with Gly863 and SH group did a π–sulphur interaction with His909 (Fig. 3) while compound S4, which had an IC_50_ of 14.94 nM showed only an additional hydrogen bonding with Tyr907 through the amino group of the pyrimidine ring (Fig. 5) and compound S5, which had an IC_50_ of 11.07 nM, showed an additional π–sulphur interaction with Tyr907.

**Table 3:** CDOCKER interaction energy and key amino acids involved in the interaction of the synthesized compounds

Compound	IC_50_ (nM)	CDOCKER interaction energy (kcal mol^−1^)	Key amino acids involved in the interaction
S1	49.06 ± 2.32	53.1	GLY863 H-bond with C=O of ring
			GLY863 H-bond with NH of ring
			Ser904 H-bond with C=O of ring
			HIS862 π–π with pyrimidinedione
			TYR896 π–π with thiophene ring
			TYR907 π–π with pyrimidinedione
S2	4.06 ± 0.18	57.6	GLY863 H-bond with C=O of ring
			GLY863 H-bond with NH of ring
			Gly863 H-bond with amino group
			Ser904 H-bond with C=O of ring
			HIS862 π–π with pyrimidinedione
			TYR896 π–π with thiophene ring
			TYR907 π–π with pyrimidinedione
			His 909 π–sulphur with SH
S3	71.41 ± 4.12	45.6	GLY863 H-bond with C=O of ring
			GLY863 H-bond with NH of ring
			Ser904 H-bond with C=O of ring
			ARG878 H-bond with NO_2_
			HIS862 π–π with pyrimidinedione
			TYR896 π–π with thiophene ring
			TYR907 π–π with pyrimidinedione
S4	14.94 ± 1.03	54.1	GLY863 H-bond with C=O of ring
			GLY863 H-bond with NH of ring
			Ser904 H-bond with C=O of ring
			TYR907 H-bond with amino group
			HIS862 π–π with pyrimidinedione
			TYR896 π–π with thiophene ring
			TYR907 π–π with pyrimidinedione
S5	11.07 ± 0.76	55.7	GLY863 H-bond with C=O of ring
			GLY863 H-bond with NH of ring
			Ser904 H-bond with C=O of ring
			HIS862 π–π with pyrimidinedione
			TYR896 π–π with thiophene ring
			TYR907 π–π with pyrimidinedione
			TYR907 π–sulphur with =S
S6	114.95 ± 7.22	49.4	GLY863 H-bond with C=O of ring
			GLY863 H-bond with NH of ring
			Ser904 H-bond with C=O of ring
			HIS862 π–π with pyrimidinedione
			TYR896 π–π with thiophene ring
			TYR907 π–π with pyrimidinedione
S7	3.61 ± 0.15	58.9	GLY863 H-bond with C=O of ring
			GLY863 H-bond with NH of ring
			Ser904 H-bond with C=O of ring
			HIS862 π–π with pyrimidinedione
			TYR896 π–π with thiophene ring
			TYR907 π–π with pyrimidinedione
			Ser 864 C–H with C=O
S8	15.79 ± 0.86	54.2	GLY863 H-bond with C=O of ring
			GLY863 H-bond with NH of ring
			ARG878 H-bond with NO_2_
			Ser904 H-bond with C=O of ring
			HIS862 π–π with pyrimidinedione
			TYR896 π–π with thiophene ring
			TYR907 π–π with pyrimidinedione
S9	48.55 ± 3.25	52.4	GLY863 H-bond with C=O of ring
			GLY863 H-bond with NH of ring
			Ser904 H-bond with C=O of ring
			HIS862 π–π with pyrimidinedione
			TYR896 π–π with thiophene ring
			TYR907 π–π with pyrimidinedione
S10	16.16 ± 0.88	53.2	GLY863 H-bond with C=O of ring
			GLY863 H-bond with NH of ring
			Ser904 H-bond with C=O of ring
			HIS862 π–π with pyrimidinedione
			TYR896 π–π with thiophene ring
			TYR907 π–π with pyrimidinedione
			TYR896 π–alkyl with methyl
Olaparib	5.77 ± 0.26	57.5	GLY863 H-bond with C=O of phthalazinone
			SER904 H-bond with C=O of phthalazinone
			TYR896 H-bond with C=O of linker
			ARG878 H-bond with C=O of piperazine
			TYR896 π–π with fluorophenyl
			TYR907 π–π with phthalazinone
			HIS862 π–+ with phthalazinone

Compound S7, which had an IC_50_ of 3.61 nM, showed an additional carbon–hydrogen interaction with Ser864 also its phenyl ring laid in a deep hydrophobic pocket lined with the side chain of Asn868, Ile872 and Leu877 within the binding site (Fig. 4). Compound S8, which had an IC_50_ of 15.79 nM, showed an additional hydrogen bond with Arg878 through the nitro group while compound S10, which had an IC_50_ of 16.16 nM, showed an additional π–alkyl interaction with Tyr896 through its methyl group (Fig. 5).

### 3.2 Structure–activity relationship (SAR)

Based on the observed pharmacological and molecular docking data we can conclude that presence of pyrano[2,3-*d*]pyrimidine 2,4 dione scaffold is important for interactions with the amino acids present in the NI site of the enzyme, addition of a fused heterocycle resulted in extra interactions with the enzyme and greatly enhanced the activity, also presence of hydrophobic substituent on the ring was favorable due to interaction with the AD site of the enzyme.

### 3.3. *In silico* ADMET study

**Fig. 6:**
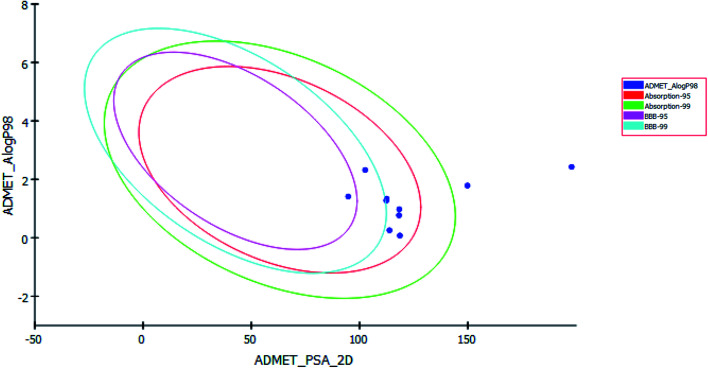
ADMET plot for the newly synthesized compounds.

Pharmacokinetics properties of synthesized compounds were predicted using ADMET protocol in Accelrys Discovery Studio 4.1 software. The results of the ADMET study are presented as ADMET-Plot, which is a 2D plot drawn using calculated PSA_2D and *A* log P98 properties (Fig. 6).

In BBB plot, most of the compounds except S5 and S7 were fallen outside the 99% ellipse. Hence, these compounds may not be able to penetrate the blood brain barrier; hence the chances of CNS side effects are predicted to be low.

In HIA plot, most of compounds fell inside the 99% ellipse, thus estimated to have good human intestinal absorption except for S3 and S8 which showed poor absorption. Aqueous solubility level of most of the compounds was found to be 3 or 2 which indicates low aqueous solubility. The hepatotoxicity level of all compounds was 1. Hence the compounds are predicted to possess hepatotoxicity. Further experimental studies are required to determine the hepatotoxic dose levels. Most of the compounds are predicted as noninhibitors of CYP2D6. Hence the side effects (*i.e.* liver dysfunction) are not expected upon administration of these compounds.

The plasma protein-binding model predicts whether a compound is likely to be highly bound to carrier proteins in the blood. There is diversity in the synthesized compounds regarding their ability to bind to plasma proteins.

PSA is a key property that has been linked to drug bioavailability. Thus, passively absorbed molecules with PSA > 140 are thought to have low bioavailability. Most of the synthesized compounds have PSA ranging from 94.77–118.63, thus, they are predicted to present good passive oral absorption except for compounds S3 and S8 which had PSA more than 140. The calculated parameters from the ADMET study are tabulated in Table 4 (ESI†).

**Table 4:** Computer aided ADMET screening of the synthesized compounds

CPD ID	BBB_Lev^a^	Absorp_Lev^b^	AQ SOlLEV^c^	Hepatox^d^	Hepatox Prob^e^	CYP 2D6^f^	CYP 2D6Prob^g^	PPB_Lev^h^	*A* log P98^i^	ADEM_PSA_2D^j^
1	4	0	3	1	0.748	0	0.128	0	0.077	118.62
2	4	0	2	0	0.47	0	0.287	0	0.978	118.33
3	4	3	1	1	0.94	0	0.346	0	2.247	197.94
4	4	0	3	1	0.913	0	0.485	2	0.77	118.21
5	3	0	2	1	0.854	0	0.306	0	1.413	94.77
6	4	0	3	1	0.821	0	0.445	1	0.258	113.82
7	3	0	2	1	0.966	0	0.495	2	2.321	102.61
8	4	2	2	1	0.88	0	0.445	2	1.787	149.83
9	4	0	3	1	0.503	1	0.514	0	1.276	112.34
10	4	0	3	1	0.9	0	0.326	0	1.341	112.48

a Blood brain barrier level; 4 = undefined, 2 = medium penetration, 1 = high penetration.

b Absorption level; 3 = very low absorption, 2 = low absorption, 1 = moderate, 0 = good absorption.

c Aqueous solubility level; 4 = optimal, 3 = good, 2 = low solubility, 1 = very low but soluble, 0 = extremely low.

d Hepatotoxicity level; 1 = toxic, 0 = nontoxic.

e Hepatotoxicity probability.

f CYP2D6 inhibition; 1 = likely to inhibit, 0 = non inhibitor.

g Cyp2D6 inhibition probability.

h Plasma protein binding; 2 = more than 95%, 1 = more than 90%, 0 = less than 90%.

i Lipophilicity descriptor; compounds must have log *p* value not greater than 5.0 to attain a reasonable probability of being well absorbed.

j Polar surface area.

## 4. Conclusion

In conclusion, a series of novel pyrano[2,3-*d*] pyrimidine 2,4 dione analogues were designed and synthesized based on the characteristics of the catalytic domain in PARP-1. These compounds were evaluated for their PARP-1 enzyme inhibitory activity and cellular inhibitory against MCF7 and HCT116 human cancer cell lines. Compounds S2 and S7 showed a higher potency than the reference Olaparib indicating that the presence of an extra fused heterocycle ring enhanced the activity. Finally, a molecular docking study was performed to investigate the probable interactions with the PARP-1 enzyme.

## 5. Experimental section

### 5.1. Chemistry

Melting points of all the new compounds are listed uncorrected and were measured by a Reichert Thermovar apparatus. Yields registered are of the new compounds. The IR spectra were determined using Perkin-Elmer spectrometer (KBr disc), model 1720 FTIR. ^1^H-NMR, and ^13^C-NMR spectra were done using a Bruker AC-300 or DPX-300 spectrometers. Chemical shifts were reported in *δ* scale (ppm) using TMS as a reference standard and the coupling constants *J* values are given in Hz. The progress of the reactions was determined using TLC aluminum silica gel plates 60 F245. IR, the analysis (^1^H-NMR, ^13^C-NMR and elemental analyses) were done on the Main Chemical Warfare Laboratories, Chemical Warfare Department, Egypt.

#### 5.1.1 Synthesis of 7-amino-2,4-dioxo-5-(thiophen-2-yl)-2,3,4,5-tetrahydro-1*H*-pyrano[2,3-*d*]pyrimidine-6-carbonitrile (1)

A mixture of thiophen-2-carbaldehyde (1.12 g, 10 mmol), malononitrile (6.6 g, 100 mmol) barbituric acid (12.8 g, 0.1 mol) and triethylamine (2–3 drops) in a solution of water: ethanol (1 : 1 ratio) 30 ml : 30 ml were heated under reflux for 2 h. The solid separated and collected after concentration and cooling of the solvent at room temperature, dried and crystallized from butanol to give 1 as yellow crystals; mp: 295–298 °C, yield 95%. IR (KBr) 3449.18 cm^−1^ (*ν*_NH2_), 3203.12, 3148.84 cm^−1^ (*ν*_NH_), 2070.14 cm^−1^ (*ν*_CN_), 1751.88, 1694.47 cm^−1^ (*ν*_C=O_). ^1^H-NMR (DMSO-d_6_): 7.32, 7.34 (m, 3H, thiophen), 8.71 (s, 2H, D_2_O exchangeable NH_2_) 7.93, 11.27 (s, 1H,D_2_O exchangeable NH). Anal. calculated for C_12_H_8_N_4_O_3_S (288.28): C, 50.00; H, 2.80; N, 19.43; S, 11.12. Found: C, 49.98; H, 2.69; N, 19.41; S, 11.06.^18,19^

#### 5.1.2 Synthesis of 4-amino-2-mercapto-10-(thiophen-2-yl)-6,10-dihydro-7*H*-pyrano[2,3-*d*:5,6-*d*']dipyrimidine-7,9(8*H*)-dione (2)

A mixture of compound (1) (0.78 g, 25 mmol) with thiourea (0.18 g, 30 mmol) was fused for 2 h, then dissolved in a solution of sodium hydroxide (10%) with stirring for another 2 h, then neutralized by HCl to obtain solid product which was filtered off, washed with water, dried and recrystallized from acetic acid to give 2. Yields 86%, mp 170–172 °C, yellow powder; IR (KBr, *ν*/cm^−1^): 3420.87, 3281.06 cm^−1^ (*ν*_NH2_); 3256.68, 3163.37 cm^−1^ (2*ν*_NH_), 2687.10 cm^−1^ (*ν*_SH_), 1752.22, 1694.82 cm^−1^ (2 *ν*_C=O_). ^1^H-NMR (DMSO-d_6_, 300 MHz): *δ* 4.03 (singlet, 1H, pyran CH),7.029 (singlet, 2H, NH_2_, D_2_O exchangeable), 7.341–7.319 (m, 3H, thiophen), 9.594 ppm (s, 1H, NH, D_2_O exchangeable), 10.005 ppm (s, 1H, NH, D_2_O exchangeable), 11.271 ppm (s, 1H, SH, D_2_O exchangeable). ^13^C-NMR (DMSO-d_6_, 300 MHz) *δ* (ppm): 39.31 (CH), 112.02 (C=C); 19.5; 112.02; 128.83 (2CH); 136.76; 142.57; 146.16 (2 C–O), 146.29 (C–N); 150.69 (C–NH_2_); 163.48, 163.97 (C=O), 184.27 (C–SH), Anal. calculated for C_13_H_9_N_5_O_3_S_2_ (347.37): C, 44.95; H, 2.61; N, 20.16; S, 18.46, found: C, 44.85; H, 2.83; N, 19.44; S, 17.37.

#### 5.1.3 Synthesis of 3-amino-1-(2,5-dinitrophenyl)-9-(thiophen-2-yl)-5,9-dihydropyrazolo[3′,4':5,6]pyrano[2,3-*d*]pyrimidine-6,8(1*H*,7*H*)-dione (3)

A mixture of 7-amino-2,4-dioxo-5-(thiophen-2-yl)-2,3,4,5-tetrahydro-1*H*-pyrano[2,3-*d*]pyrimidine-6-carbonitrile (1) (0.78 g, 25 mmol) and 2,4 dinitrophenylhydrazine (0.49 g, 25 mmol) in 1,4-dioxane (30 ml) was refluxed in water bathe for 3 h, cooled, filtered off, dried and recrystallized from methanol to give 3. Yields 86%, mp 225 °C, orange powder; IR (KBr, *ν*/cm^−1^): 3420.17, 3365.15 cm^−1^ (*ν*_NH_2__), 3256.68, 3163.37 cm^−1^ (2*ν*_NH_); 1752.22, 1694.82 cm^−1^ (*ν*_C=O_), 1656.12 cm^−1^ (*ν*_C=C_), 1589.47 cm^−1^ (*ν*_C=N_), 1468.39 cm^−1^ (*ν*_C=S_). ^1^H-NMR (300 MHz, DMSO-d_6_): *δ* 3.82 (singlet, 1H, puran CH), 6.69–7.21 (m, 3H, thiophen)_,_ 7.94 (d, 1H, *j* = 7.2, ArH), 8.01 (d, 1H, *j* = 7.2, ArH) 8.2 (s, 1H, ArH), 9.42, 9.46 ppm (s, 2H, NH_2_, D_2_O exchangeable), 9.12, 10.57 ppm (s, 1H, 2NH, D_2_O exchangeable). ^13^C-NMR (DMSO-d_6_, 300 MHz) *δ* (ppm): 31.72 (CH), 112.02 (C), 116.73, 123.50, 123.54 (CH), 128.71, 128.82, 130.27 (CH); 130.43 (C–O); 132.19 (CH); 136.77 (C–N); 130.30, 138.85 (C=C), 142.57, 144.60 (C–NO_2_), 150.69 (C–NH), 159.71 (C–NH_2_), 163.96 (C=O). Anal. calculated for C_18_H_11_N_7_O_7_S (469.39): C, 46.06; H, 2.36; N, 20.89; S, 6.83, found: C, 45.85; H, 2.43; N, 19.94; S, 7.02.

#### 5.1.4 Synthesis of 4-amino-10-(thiophen-2-yl)-6,10-dihydro-7*H*-pyrano[2,3-*d*:5,6-*d*']dipyrimidine-7,9(8*H*)-dione (4)

A solution of 7-amino-2,4-dioxo-5-(thiophen-2-yl)-2,3,4,5-tetrahydro-1*H*-pyrano[2,3-d]pyrimidine-6-carbonitrile (1) (1.56 g, 5 mmol) and formamide (20 ml) for was refluxed for 2 h, then cooled, poured into crushed-ice, filtered off, dried and recrystallized from methanol to give 4. Yields 76%, mp over 300 °C, black powder; IR (KBr, *ν*/cm^−1^): 3470.68, 3249.52 cm^−1^ (*ν*_NH2_); 3214.28, 3111.74 cm^−1^ (2*ν*_NH_), 1751.15, 1689.78 cm^−1^ (*ν*_C=O_), 1557.04 cm^−1^ (*ν*_C=N_). ^1^H-NMR (300 MHz, DMSO-d_6_): *δ* 4.10 (singlet, 1H, puran CH), 6.90–7.377 (m, 3H, thiophen), 7.96 (S, 2H, pyrimidine), 9.121, 9.426 (s, 2H, NH_2_, D_2_O exchangeable), 6.626, 10.575 ppm (s, 1H, NH, D_2_O exchangeable). Anal. calculated for C_13_H_9_N_5_O_3_S (315.31): C, 49.52; H, 2.88; N, 22.21; S, 10.17, found: C, 49.85; H, 2.83; N, 21.94; S, 10.09.

#### 5.1.5 Synthesis of 10-(thiophen-2-yl)-2,4-dithioxo-1,2,3,4,6,10-hexahydro-7*H*-pyrano[2,3-*d*:5,6-*d*']dipyrimidine-7,9(8*H*)-dione (5)

A mixture of 7-amino-2,4-dioxo-5-(thiophen-2-yl)-2,3,4,5-tetrahydro-1*H*-pyrano[2,3-d]pyrimidine-6-carbonitrile (1) (1.56 g, 5 mmol) and C_2_S (10 ml) in dry pyridine (25 ml) was heated under reflux in water bath for 6 h, cooled, poured into ice-water, acidified with glacial acetic acid then filtered, dried and recrystallized from ethanol to give 5. Yields 85%, mp over 300 °C, greenish yellow powder; IR (KBr, *ν*/cm^−1^): 3470.68, 3249.52, 3214.28, 3111.74 cm^−1^ (4*ν*_NH_); 1751.16, 1689.78 cm^−1^ (2*ν*_C=O_), 1658.59 cm^−1^ (*ν*_C=C_). ^1^H-NMR (300 MHz, DMSO-d_6_): *δ* 3.99 (singlet, 1H, puran CH), 7.30–7.32 (m, 3H, thiophen), 7.34, 11.236, 11.277 ppm (s, 1H, 3NH, D_2_O exchangeable), ^13^C-NMR (DMSO-d_6_, 300 MHz) *δ* (ppm): 39.33 (CH), 112.02 (C), 128.83, 142.57 (CH); 136.77, 146.16 (2CH), 146.28, 150.69 (C–NH), 163.48 (2C=O), 163.96 (2C=S). Anal. calculated for C_13_H_8_N_4_O_3_S_3_ (364.42): C, 42.85; H, 2.21; N, 15.37; S, 26.40, found: C, 42.65; H, 2.18; N, 15.44; S, 26.38.

#### 5.1.6 Synthesis of 6-(methoxyamino)-2,4-dioxo-5-(thiophen-2-yl)-2,3,4,5-tetrahydro-1*H*-pyrano[2,3-*d*]pyrimidine-7-carbonitrile (6)

A mixture of 7-amino-2,4-dioxo-5-(thiophen-2-yl)-2,3,4,5-tetrahydro-1*H*-pyrano[2,3-*d*]pyrimidine-6-carbonitrile (1) (1.56 g, 5 mmol) and acetic anhydride (20 ml) was refluxed for 4 h. The solid separated and collected after concentration and cooling of the solvent to room temperature, dried and crystallized from butanol to give 6 as yellow powder; mp: over 300 °C, yield 90%. IR (KBr) 3468.7, 3254.05 and 3196.21 cm^−1^ (3*ν*_NH_), 2927.27 cm^−1^ (*ν*_CH3_), 2074.78 cm^−1^ (*ν*_CN_), 1730.52, 1690.42 cm^−1^ (2*ν*_C=O_). ^1^H-NMR (300 MHz, DMSO-d_6_): *δ* 3.38 (s, 1H, OCH_3_), 3.99 (singlet, 1H, puran CH), 2.47 (s, NH, D_2_O exchangeable), 7.32–8.54 (m, 3H, thiophen), 11.23, 11.27 (s, 2H, NH, D_2_O exchangeable). Anal. calculated for C_13_H_10_N_4_O_4_S (318.31): C, 49.05; H, 3.17; N, 17.60; S, 10.07. Found: C, 49.55; H, 3.18; N, 17.52.

#### 5.1.7 Synthesis of 3-phenyl-10-(thiophen-2-yl)-2-thioxo-2,3,6,10-tetrahydro-1*H*-pyrano[2,3-*d*:5,6-*d*']dipyrimidine-4,7,9(8*H*)-trione (7)

A mixture of 7-amino-2,4-dioxo-5-(thiophen-2-yl)-2,3,4,5-tetrahydro-1*H*-pyrano[2,3-*d*]pyrimidine-6-carbonitrile (1) (1.56 g, 5 mmol) and phenyl isothiocyanate (0.67 g, 5 mmol) in dry pyridine (20 ml) was heated under reflux for 4 h. After cooling, the reaction mixture was diluted with ice cold water, acidified to litmus paper with glacial acetic acid, filtered and crystallized from butanol to give 7 as white powder; mp: 235–237 °C, yield 70%. IR (KBr): 3464.92, 3329.11, 3062.09 cm^−1^ (3*ν*_NH_), 1717, 1694.58 cm^−1^ (2*ν*_C=O_). ^1^H-NMR (300 MHz, DMSO-d_6_): *δ* 3.99 (singlet, 1H, puran CH), 2.066, 6.89 (s, 2H, NH, D_2_O exchangeable), 6.90–6.94 (m, 3H, thiophen), 7.22–7.46 (m, 5H, ArH), 9.19 (s, 1H, NH, D_2_O exchangeable). Anal. calculated for C_19_H_12_N_4_O_4_S_2_ (424.45): C, 53.76; H, 2.85; N, 13.20; S, 15.11. Found: C, 53.55; H, 2.76; N, 12.82, S, 14.91.

#### 5.1.8 Synthesis of 6-amino-8-(4-nitrophenyl)-5-(thiophen-2-yl)-1,5,5*a*,9*a*-tetrahydro-2*H*-pyrido[3′,2':5,6]pyrano[2,3-*d*]pyrimidine-2,4(3*H*)-dione (8)

A mixture of 7-amino-2,4-dioxo-5-(thiophen-2-yl)-2,3,4,5-tetrahydro-1*H*-pyrano[2,3-*d*]pyrimidine-6-carbonitrile (1) (1.56 g, 5 mmol), *p*-nitroacetophenone (0.82 g, 5 mmol) and 2–3 drops of triethylamine in 1,4-dioxane (30 ml) was heated under reflux for 5 h then cooled and poured into ice-water, filtered off, dried and recrystallized from 1,4-dioxane to give 8 as orange crystals; mp: 130 °C, yield 82%. IR (KBr): 3201.92 cm^−1^ (*ν*_NH_2__), 3147.46, 3108.03 cm^−1^ (2*ν*_NH_), 1751.88, 1688.98 cm^−1^ (2*ν*_C=O_), 1552.69 cm^−1^ (*ν*_NO_2__). ^1^H-NMR (300 MHz, DMSO-d_6_): *δ* 3.85 (s, 1H, puran CH), 6.95 (s, 1H, NH, D_2_O exchangeable), 7.11–7.34 (m, 3H, thiophen), 7.72 (s, 1H, NH, D_2_O exchangeable), 7.98 (s, 1H, NH_2_, D_2_O exchangeable), 8.18–8.36 (d, 4H, ArH), ^13^C-NMR (DMSO-d_6_, 300 MHz) *δ* (ppm): 39.33 (CH), 40.59 (C); 124.39, 125.13, 129.08, 129.09 (CH); 126.73, 129.75, 129.97, 130.22 (CH); 114.49, 129.08 (C=C); 130.39 (C=N); 136.08 (C–NO_2_), 141.60 (C–NH), 143.05 (C=O), 146.08 (C–NH_2_). Anal. calculated for C_20_H_15_N_5_O_5_S (437.43): C, 54.92; H, 3.46; N, 16.01; S, 7.33. Found: C, 54.23; H, 3.04; N, 15.099; S, 6.82.

#### 5.1.9 Synthesis of (*E*)-ethyl *N*-(6-cyano-2,4-dioxo-5-(thiophen-2-yl)-2,3,4,5-tetrahydro-1*H*-pyrano[2,3-*d*]pyrimidin-7-yl)formimidate (9)

A mixture of 7-amino-2,4-dioxo-5-(thiophen-2-yl)-2,3,4,5-tetrahydro-1*H*-pyrano[2,3-*d*] pyrimidine-6-carbonitrile (1) (1.56 g, 5 mmol) and (30 ml, 5 mmol) triethylorthoformate was refluxed for 7 h. The solid separated and collected after concentration and cooling of the solvent to room temperature was dried and crystallized from butanol to give 9 as yellow crystals; mp: 293–295 °C, yield 90%. IR (KBr): 3199.9, 3147.11 cm^−1^ (2*ν*_NH_), 2069.45 cm^−1^ (*ν*_CN_), 1751.42, 1665.32 cm^−1^ (*ν*_C=O_). ^1^H-NMR (300 MHz, DMSO-d_6_): *δ* 1.036 (t, 3H, CH_3_), 3.25 (q, 2H, CH_2_), 3.69 (singlet, 1H, puran CH), 7.32–7.34 (m, 3H, thiophen), 8.71 (s, 1H, NH, D_2_O exchangeable),11.23 (s, 1H, NH, D_2_O exchangeable), ^13^C-NMR (DMSO-d_6_, 300 MHz) *δ* (ppm): 39.33 (CH), 39.96, 40.59 (C); 112.03 (C≡N); 128.82 (CH); 136.78 (C=C); 142.56 (2C–NH), 146.15 (C=N), 146.27 (2CH), 150.69, 163.48 (C–N), 163.96 (2C=O). Anal. calculated for C_15_H_12_N_4_O_4_S (344.35): C, 52.32; H, 3.51; N, 16.27; S, 9.31. Found: C, 51.78; H, 3.23; N, 16.06; S, 9.11.

#### 5.1.10 Synthesis of 6-hydroxy-8-methyl-5-(thiophen-2-yl)-1,5-dihydro-2*H*-pyrano[2,3-*d*:6,5-*d*']dipyrimidine-2,4(3*H*)-dione (10)

A mixture of 7-amino-2,4-dioxo-5-(thiophen-2-yl)-2,3,4,5-tetrahydro-1*H*-pyrano[2,3-*d*] pyrimidine-6-carbonitrile (1) (1.56 g, 5 mmol) with a hydrochloric acid and acetic acid (1 : 3) (10 : 30 ml) was refluxed for 4 h. The solid separated, collected after concentration and cooling of the solvent to room temperature, dried and crystallized from butanol to give 10 as pale-yellow powder; mp: 298 °C; yield 90%. IR (KBr): 3450 cm^−1^ (*ν*_OH_), 3125.16, 3040.10 cm^−1^ (2*ν*_NH_), 1736.04, 1686.84 cm^−1^ (*ν*_C=O_). ^1^H-NMR (300 MHz, DMSO-d_6_): *δ* 2.48 (s, 3H, CH_3_), 3.91 (singlet, 1H, pyran CH), 7.32–7.34 (m, 3H, thiophen), 11.23 (s, 1H, NH, D_2_O exchangeable), 11.27 (s, 1H, OH, D_2_O exchangeable), ^13^C-NMR (DMSO-d_6_, 300 MHz) *δ* (ppm): 39.33, 40.58 (CH); 112.02, 128.82 (CH); 136.77 (2C=C); 142.56 (C–CH_3_), 146.15 (2C–NH), 146.28 (C–O), 150.69, 163.48 (2C=O), 163.96 (C–OH). Anal. calculated for C_14_H_10_N_4_O_4_S (330.32): C, 50.91; H, 3.05; N, 16.96; S, 9.71. Found: C, 50.57; H, 2.98; N, 16.34; S, 9.10.

#### 5.1.11 Synthesis of 4-hydroxy-5-(thiophen-2-yl)-5,9-dihydro-6*H*-pyrimido[5′,4':5,6]pyrano[2,3-*d*][1,2,3]triazine-6,8(7*H*)-dione (11)

A solution of sodium nitrite (0.68 g, 10 mmol) in 10 ml of water was added to a cold solution of 7-amino-2,4-dioxo-5-(thiophen-2-yl)-2,3,4,5-tetrahydro-1*H*-pyrano[2,3-*d*]pyrimidine-6-carbonitrile (1) (1.56 g, 5 mmol) in 45 ml of a (1 : 2) mixture of HCl (15 ml) and acetic acid (30 ml) which was then stirred at room temperature for 2 h. The crude product obtained was recrystallized from acetic acid. Yields 72%, mp over 300 °C, yellow powder; IR (KBr, *ν*/cm^−1^): 3442.41 cm^−1^ (*ν*_OH_), 3199.19, 3145.80 cm^−1^ (2*ν*_NH_); 1750.04, 1693.81 cm^−1^ (2*ν*_C=O_), 1653.22 cm^−1^ (*ν*_C=N_). ^1^H-NMR (300 MHz, DMSO-d_6_): *δ* 2.066 ppm (s, 1H, OH, D_2_O exchangeable), 3.87 (singlet, 1H, puran CH), 7.324–7.347 (m, 3H, thiophen), 11.237, 11.176 (s, 1H, 2NH, D_2_O exchangeable), ^13^C-NMR (DMSO-d_6_, 300 MHz) *δ* (ppm): 39.33 (CH); 112.03 (C), 128.83, 136.77 (CH); 142.57 (C), 146.16 (2C–O), 146.27 (C–NH), 163.48 (C–OH), 150.69, 163.96 (2C=O). Anal. calculated for C_12_H_9_N_5_O_4_S (319.30): C, 45.14; H, 2.84; N, 21.93; S, 10.04, found: C, 45.04; H, 2.79; N, 20.99; S, 10.00.

### 5.2. Bioassay

#### 5.2.1 PARP inhibition assay

##### Assay procedure

PARP-1 enzyme inhibition activity was measured for using a colorimetric 96-well PARP-1 assay kit (catalog no. 80580) (BPS Bioscience), according to the manufacturer's protocol. Briefly, the histone mixture was diluted 1 : 5 with 1× PBS, 50 μl of histone solution was added to each well and incubated at 4 °C overnight. The plate was washed three times using 200 μl PBST buffer (1× PBS containing 0.05% Tween-20) per well. Liquid was removed from the wells by tapping the strip wells on clean paper towels. To each well, 200 μl of blocking buffer was added, followed by 60–90 min incubation at room temperature. Then 25 μl of PARP master mixture (consisting of 2.5 μl 10× PARP buffer + 2.5 μl 10× PARP assay mixture + 5 μl activated DNA + 15 μl distilled water) was added to each well. Olaparib was used as a positive control. 5 μl of inhibitor solution of each well labeled as “test inhibitor” was added. For the “positive control” and “blank”, 5 μl of the same solution without inhibitor was added. 1× PARP buffer was prepared by adding 1 part of 10× PARP buffer to 9 parts H_2_O (v/v), 20 μl of 1× PARP buffer was added to the wells designated as “blank”. The amount of PARP-1 required for the assay was then calculated. The reaction was initiated by adding 20 μl of diluted PARP1 enzyme to the wells designated “positive control” and “test inhibitor control”. The strip wells were incubated at room temperature for 1 hour. The strip wells were then washed three times with 200 μl PBST buffer. Then, 50 μl of 50 times diluted Streptavidin-HRP with blocking buffer was added to each well, and the strips were further incubated at room temperature for 30 min. After washing the wells three times with 200 μl PBST buffer, HRP colorimetric substrate was added to each well and the plate was incubated at the room temperature until a blue color is developed in the positive control well. Then reaction was quenched with 100 ml per well of 2 M sulfuric acid, and absorbance at 450 nm was determined. Carrier solvents were assayed as negative controls. All assays were performed in triplicate. To determine the IC_50_ value for each inhibitor, the average absorbance of each inhibitor concentration was plotted against the log of the concentration of each respective inhibitor and the IC_50_ value for each plot was obtained using computer-assisted non-linear regression analyses. Data presented are the results of at least two independent experiments done in triplicate. The results of these studies are presented as mean IC_50_ (nM).^24^

#### 5.2.2 Cell proliferation inhibition assay

We used the MTT [3-(4,5-dimethylthiazol-2-yl)-2,5-diphenyltetrazoliumbromide] (Biomatik, Wilmington, DE) assay to measure cell growth inhibition, which is based on the conversion of MTT to formazan crystals by mitochondrial dehydrogenases.^25^ Only in live cells, mitochondrial enzymes can transform MTT into insoluble formazan. After incubated with serially diluted inhibitors for 96 h, cell cultures were incubated with MTT solution (5 mg ml^−1^) for 4 h at 37 °C. Then discard the medium and DMSO was added to solubilize the reaction product formazan by shaking for 10 min. Absorbance at 492 nm was measured with a microplate reader (Thermo, MK3). Cell viability was expressed as an IC_50_ value.

### 5.3. *In silico* studies

#### 5.3.1 Molecular docking study

Molecular modeling simulation study was performed through docking of the target compounds in the binding site of PARP-1 enzyme using C-Docker protocol in Discovery Studio 4.0 Software. The X-ray crystal structure of Olaparib in complex with PARP-1 was downloaded from http://www.rscb.org/pdb (PDB ID: 5DS3) in PDB format. Computational docking is an automated computer-based algorithm designed to estimate two main terms.^26^ The first is to determine the suitable position and the orientation of certain test set molecule's pose inside the binding site in comparison to that of the X-ray crystallographic enzyme–substrate complex. The second term is the calculation of the estimated protein ligand interaction energy which is known as docking scoring.

#### 5.3.2 *In silico* ADMET study

Computer aided ADMET study was performed by using the software: Accelrys Discovery studio 2.5. These studies are based on the chemical structure of the molecule and involves the calculation of certain parameters including; atom based log P98 (*A* log P98), 2D polar surface area (ADMET 2D PSA), absorption level (Absorb LEV), aqueous solubility level (AQ SOl LEV), Blood Brain Barrier value (BBB), Blood Brain Barrier Level (BBB LEV), Cytochrome P450 2D6 (CYP2D6), Cytochrome P450 2D6 Probability (CYP PROB), hepatotoxicity (HEPATOX), Hepatotoxicity Probability (HEPATOX PROB) and plasma protein binding logarithmic level (PPB LEV).^27^

## Conflicts of interest

The authors declare no conflict of interest, financial or otherwise.

## Supplementary Material

RA-011-D0RA10321G-s001
