# Supplementary material for: Discovery of pyrano[2,3-d]pyrimidine-2,4-dione derivatives as novel PARP-1 inhibitors: design, synthesis and antitumor activity
Source: RSC Adv. 2021 Jan 22;11(8):4454–64. doi: 10.1039/d0ra10321g (PMC8694318; doi:10.1039/d0ra10321g)
Supplement: RA-011-D0RA10321G-s001 [file RA-011-D0RA10321G-s001.pdf]

Electronic Supplementary Material (ESI) for RSC Advances.
This journal is © The Royal Society of Chemistry 2021

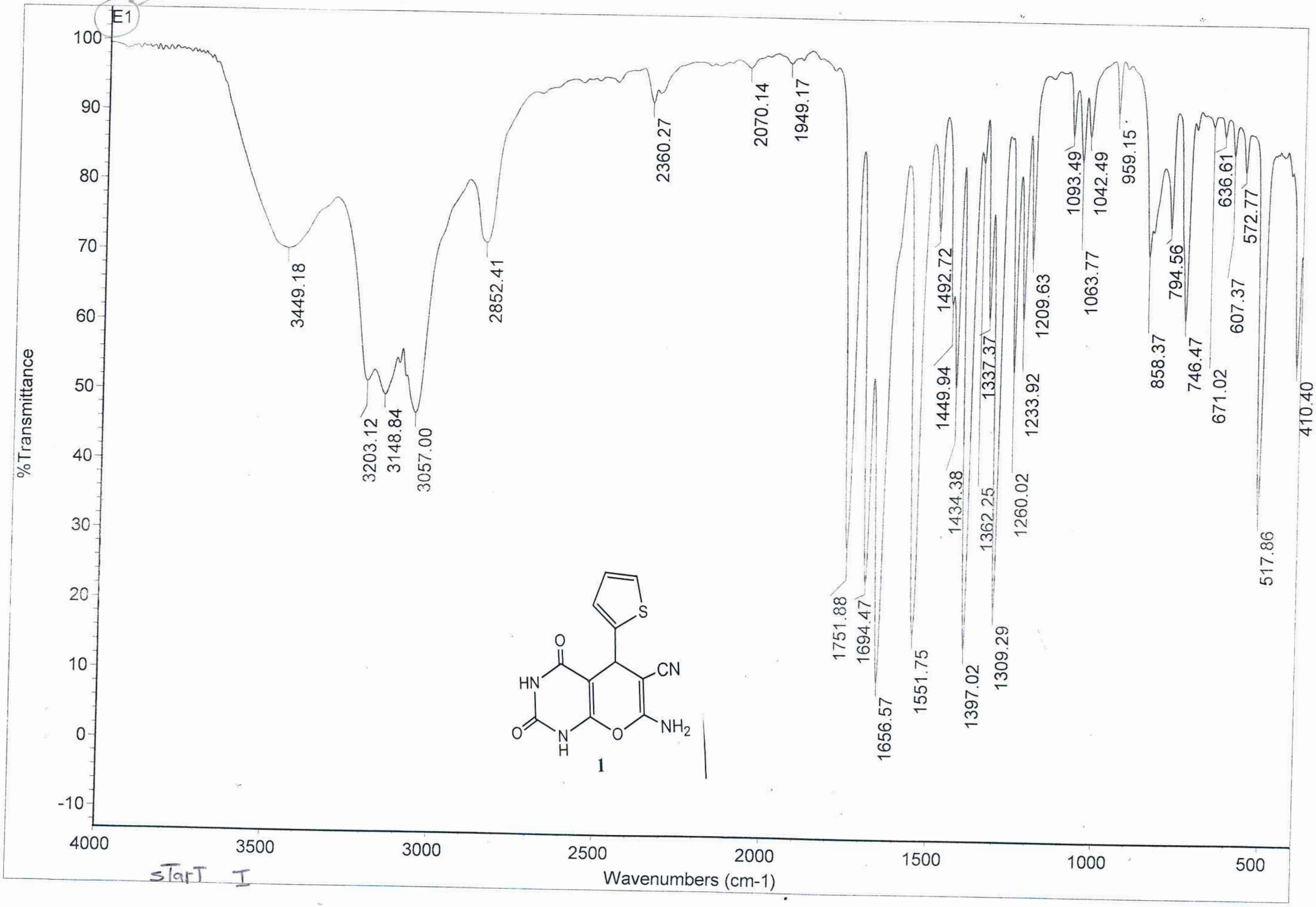

Dr_EmanHassan-E

Dr_EmanHassan-E

Sample Name	**Dr_EmanHassan-E**	Pulse sequence	**PROTON**	Temperature	**25**	Study owner	**vnmr1**
Date collected	**2018-02-18**	Solvent	**DMSO**	Spectrometer	**nmr400-mercury400**	Operator	**vnmr1**

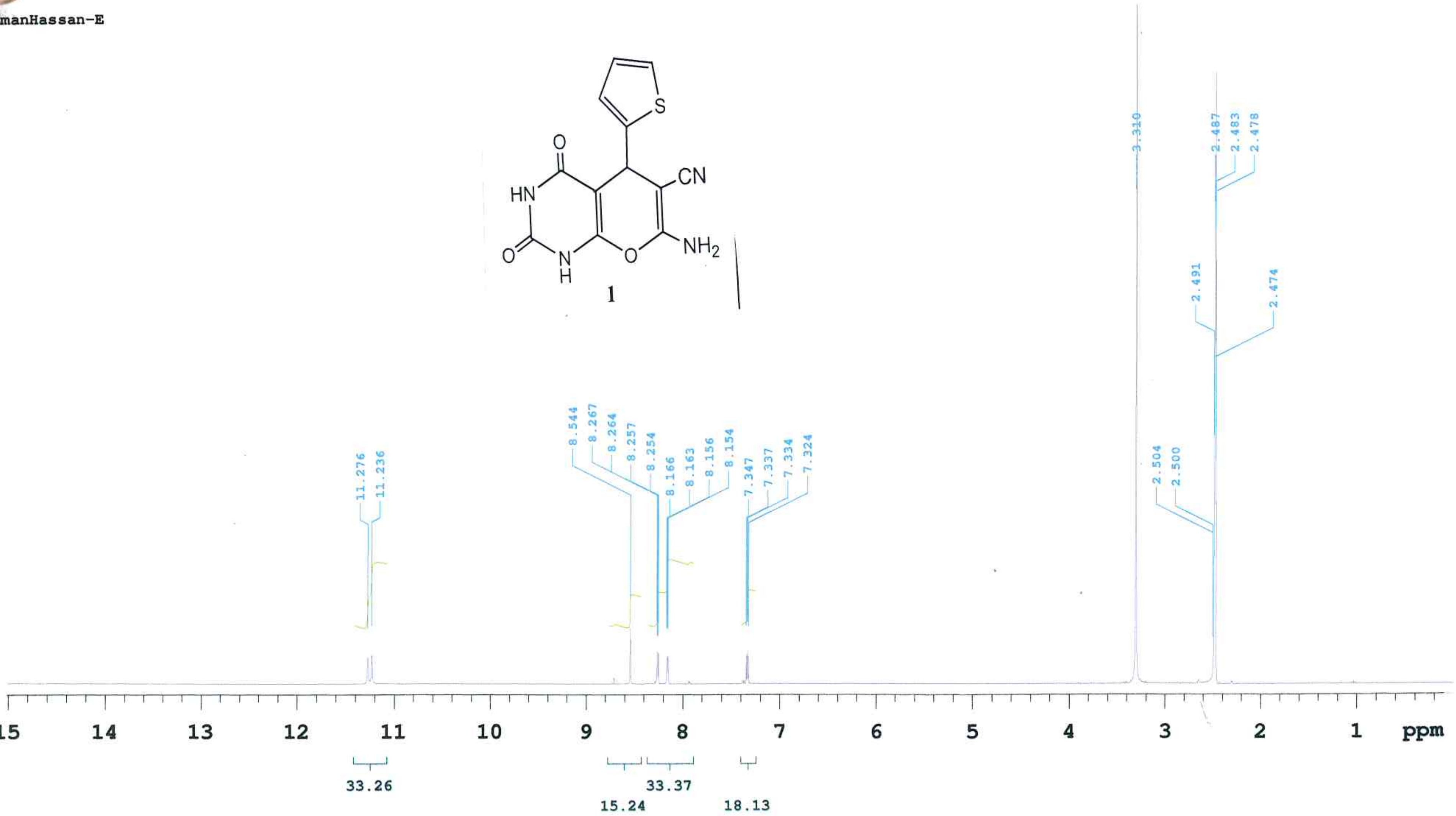

Plotname: Dr_EmanHassan-E_PROTON_01_plot02

Data file /home/data/NMRlab2018/Feb/Dr_EmanHassan-E_20180218_01/Dr_EmanHassan-E_PROTON_01

Plot date 2018-02-19

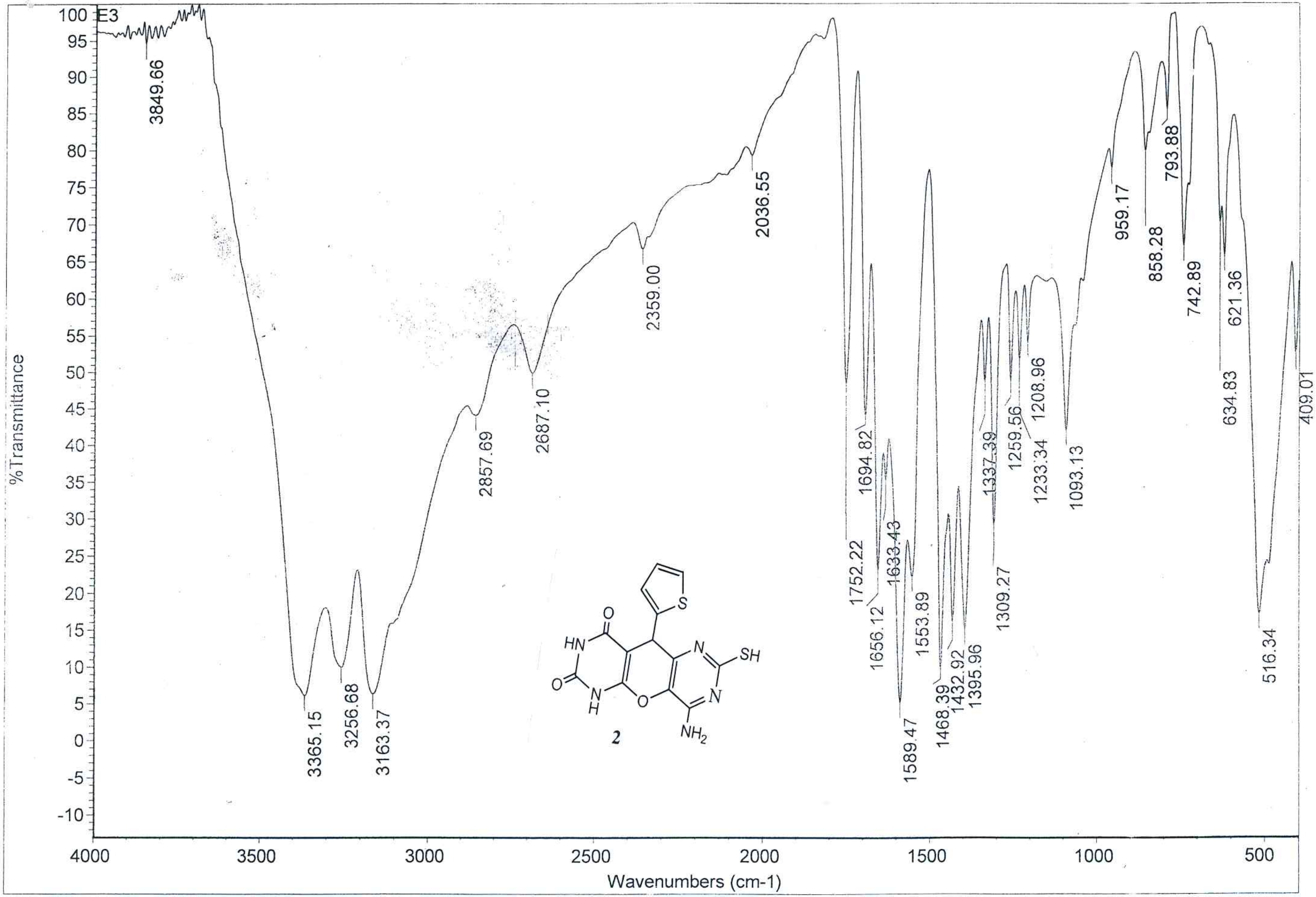
E3
%Transmittance
Wavenumbers (cm-1)
3849.66
3365.15
3256.68
3163.37
2857.69
2687.10
2359.00
2036.55
1752.22
1694.82
1656.12
1633.43
1589.47
1553.89
1468.39
1432.92
1395.96
1337.39
1309.27
1259.56
1233.34
1208.96
1093.13
959.17
858.28
793.88
742.89
634.83
621.36
516.34
409.01
HN
O
H
N
S
SH
NH2
2

① Thio urea

Dr-EmanHassan-E2

Sample Name	Dr-EmanHassan-E2	Pulse sequence	PROTON	Temperature	25	Laboratory	MODCL
Date collected	2019-02-24	Solvent	DMSO	Spectrometer	nmr400-mercury400	NMR User	

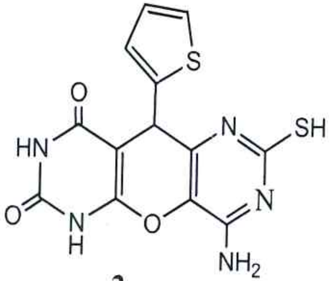

2

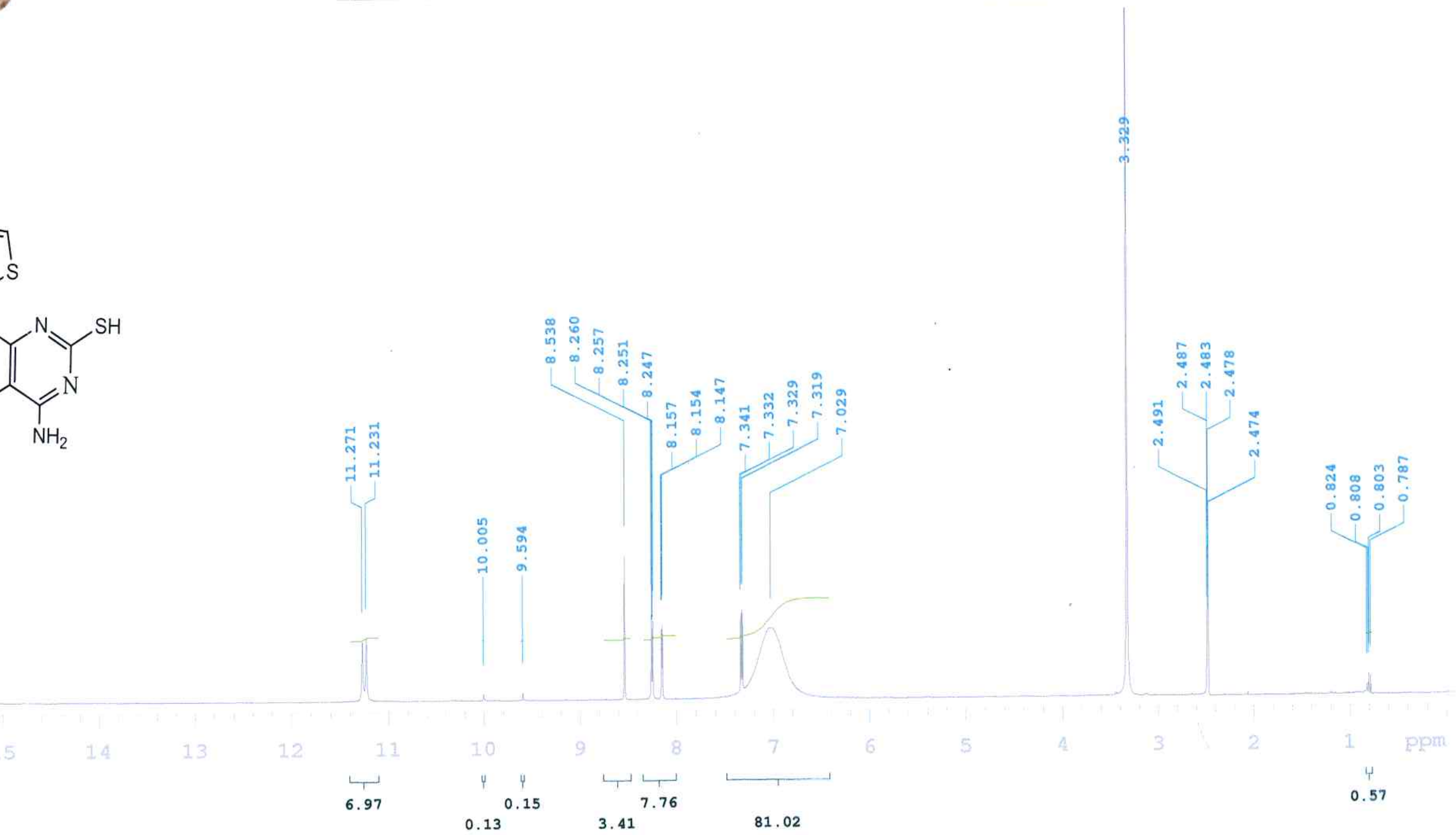

Data file /home/data/NMRlab2019/Feb/Dr-EmanHassan-E2_20190224_02/Dr-EmanHassan-E2_PROTON_01

Plot date 2019-03-09

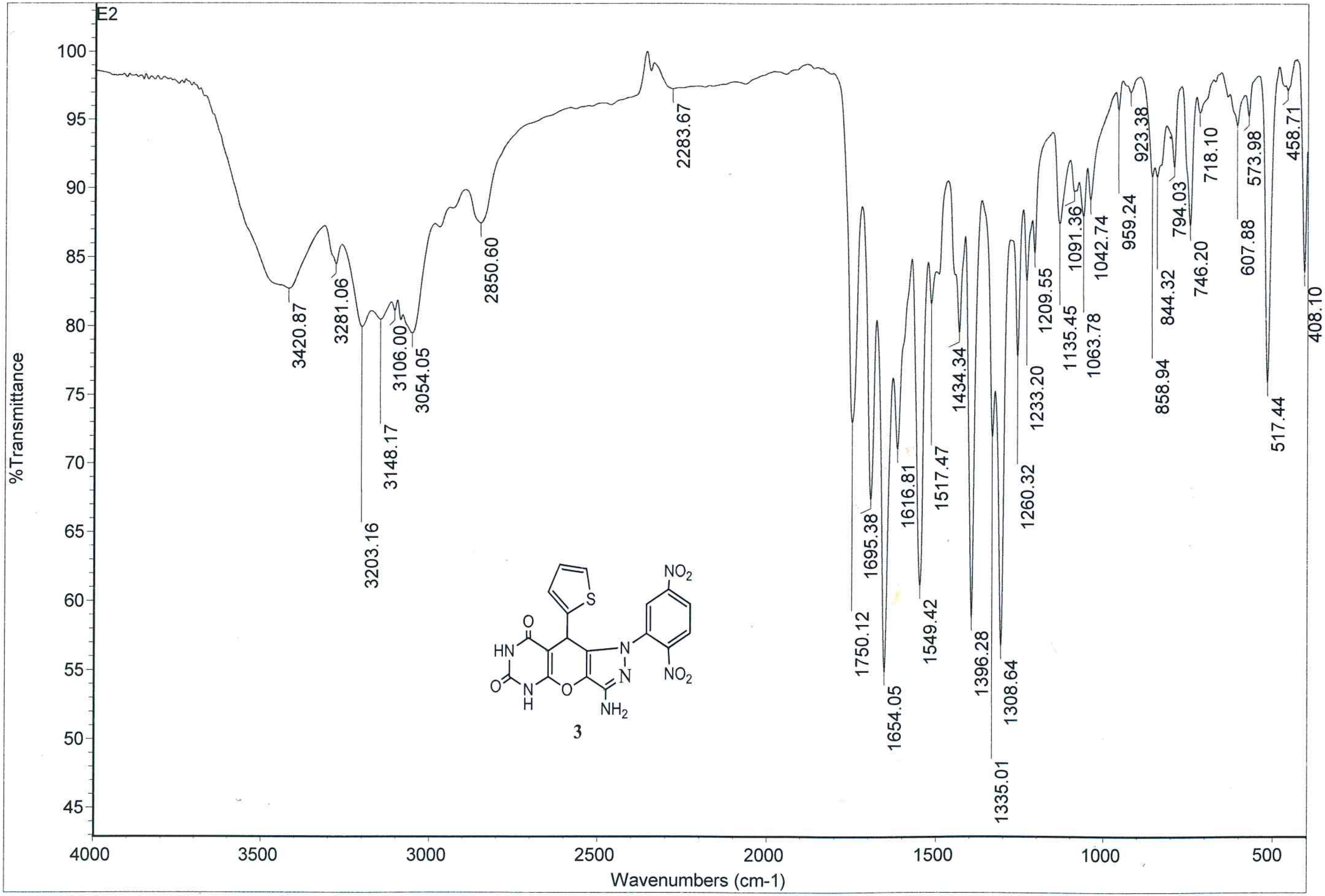
E2
%Transmittance
Wavenumbers (cm-1)
4000
3500
3000
2500
2000
1500
1000
500
100
95
90
85
80
75
70
65
60
55
50
45
3420.87
3281.06
3203.16
3148.17
3106.00
3054.05
2850.60
2283.67
1750.12
1695.38
1654.05
1616.81
1549.42
1517.47
1434.34
1396.28
1335.01
1308.64
1260.32
1233.20
1209.55
1135.45
1091.36
1063.78
1042.74
959.24
923.38
858.94
844.32
794.03
746.20
718.10
607.88
573.98
517.44
458.71
408.10
NO2
S
O
HN
N
N
NO2
O
N
H
O
NH2

3

Dr_EmanHassan-E3

Sample Name	**Dr_EmanHassan-E3**	Pulse sequence	**PROTON**	Temperature	**25**	Laboratory	**MODCL**
Date collected	**2018-09-16**	Solvent	**DMSO**	Spectrometer	**nmr400-mercury400**	NMR User	**sameeh_Albadawy**

Dr_EmanHassan-E3

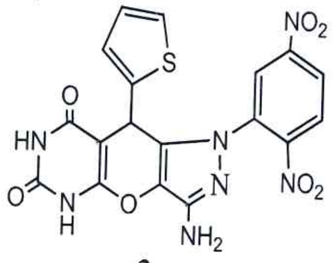

3

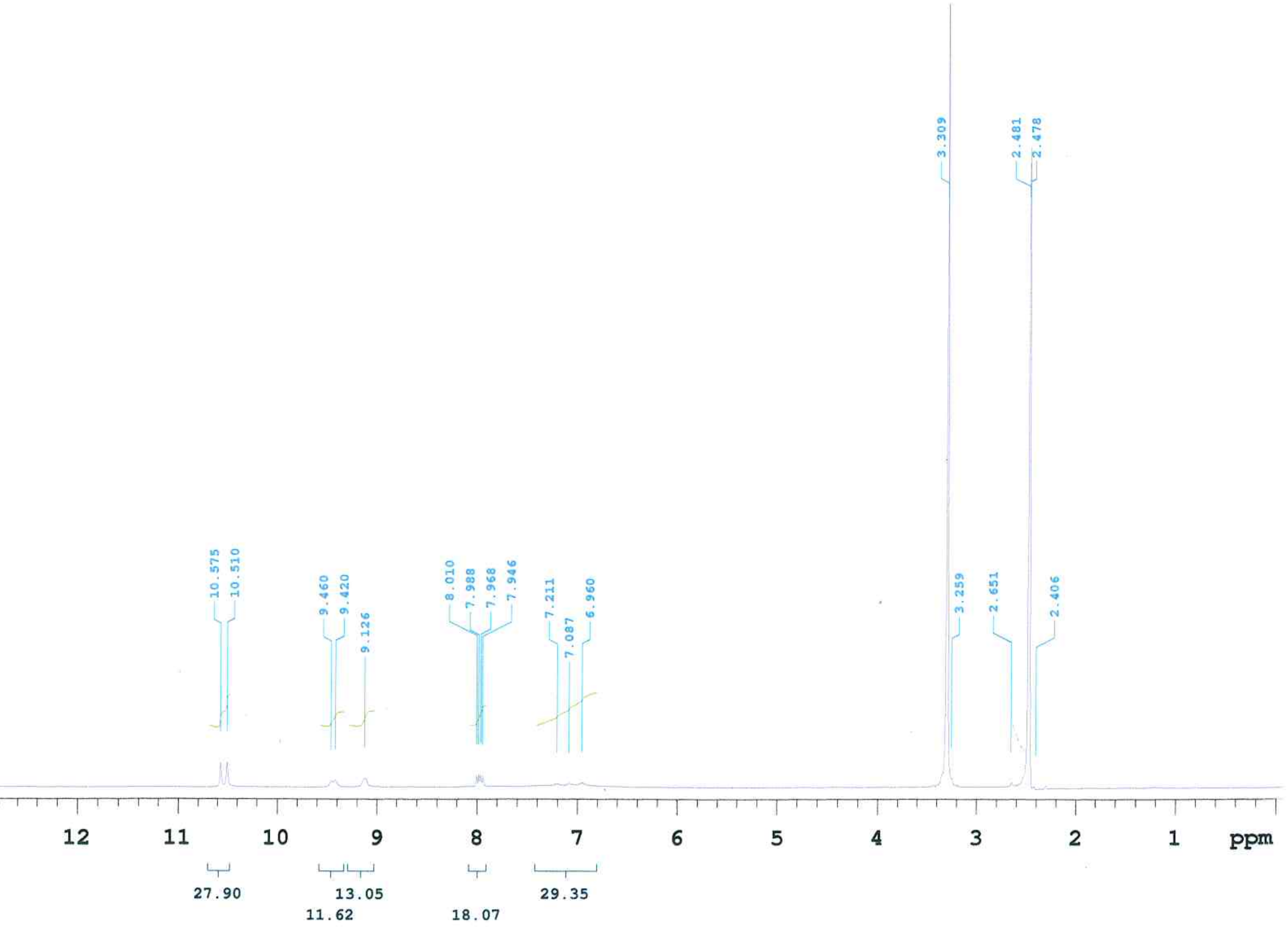

Plotname: Dr_EmanHassan-E3_PROTON_01_plot02

Data file /home/data/NMRlab2018/Sep/Dr_EmanHassan-E3_20180916_01/Dr_EmanHassan-E3_PROTON_01

Plot date 2018-09-16

Dr-EmanHassan-E3

Dr-EmanHassan-E3

Sample Name	**Dr-EmanHassan-E3**	Pulse sequence	**CARBON**	Temperature	**25**	Laboratory	**MODCL**
Date collected	**2019-02-23**	Solvent	**DMSO**	Spectrometer	**nmr400-mercury400**	NMR User	

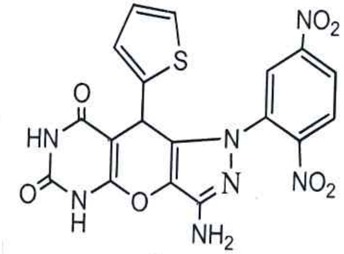

3

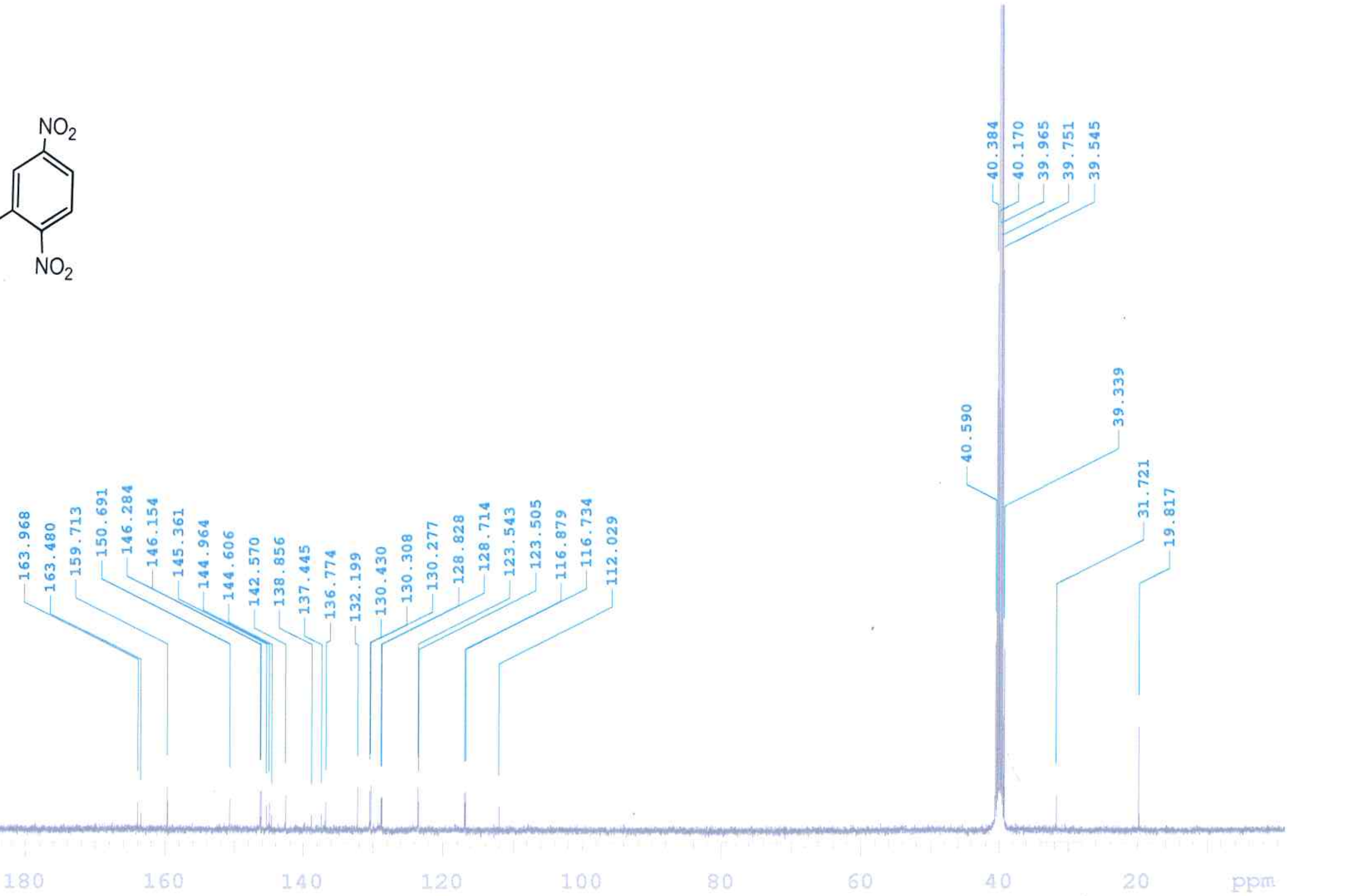

Data file /home/data/NMRlab2019/Feb/Dr-EmanHassan-E3_20190223_01/Dr-EmanHassan-E3_CARBON_01

Plot date 2019-03-09

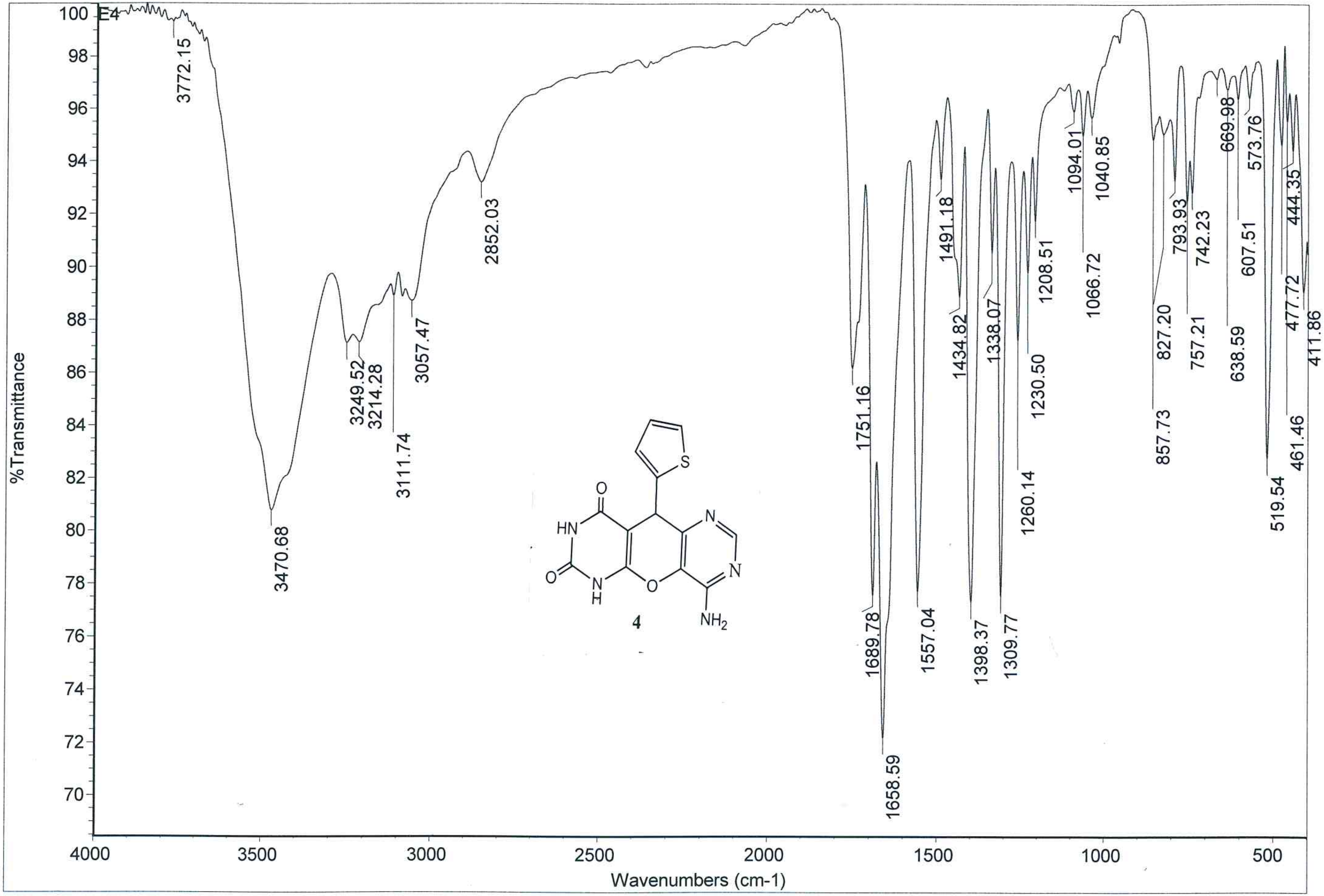

E4
%Transmittance
Wavenumbers (cm-1)
4000
3500
3000
2500
2000
1500
1000
500
100
98
96
94
92
90
88
86
84
82
80
78
76
74
72
70
3772.15
3470.68
3249.52
3214.28
3111.74
3057.47
2852.03
1751.16
1689.78
1658.59
1557.04
1491.18
1434.82
1398.37
1338.07
1309.77
1260.14
1230.50
1208.51
1094.01
1066.72
1040.85
857.73
827.20
793.93
757.21
742.23
669.98
638.59
607.51
573.76
519.54
477.72
461.46
444.35
411.86
S
O
HN
N
O
N
H
O
N
NH2
4

Dr-EmanHassan-E4

Dr-EmanHassan-E4

Sample Name	Dr-EmanHassan-E4	Pulse sequence	PROTON	Temperature	25	Laboratory	MODCL
Date collected	2019-02-24	Solvent	DMSO	Spectrometer	nmr400-mercury400	NMR User	Cap-Ahmed zaher

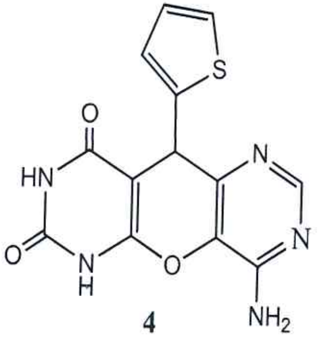

4

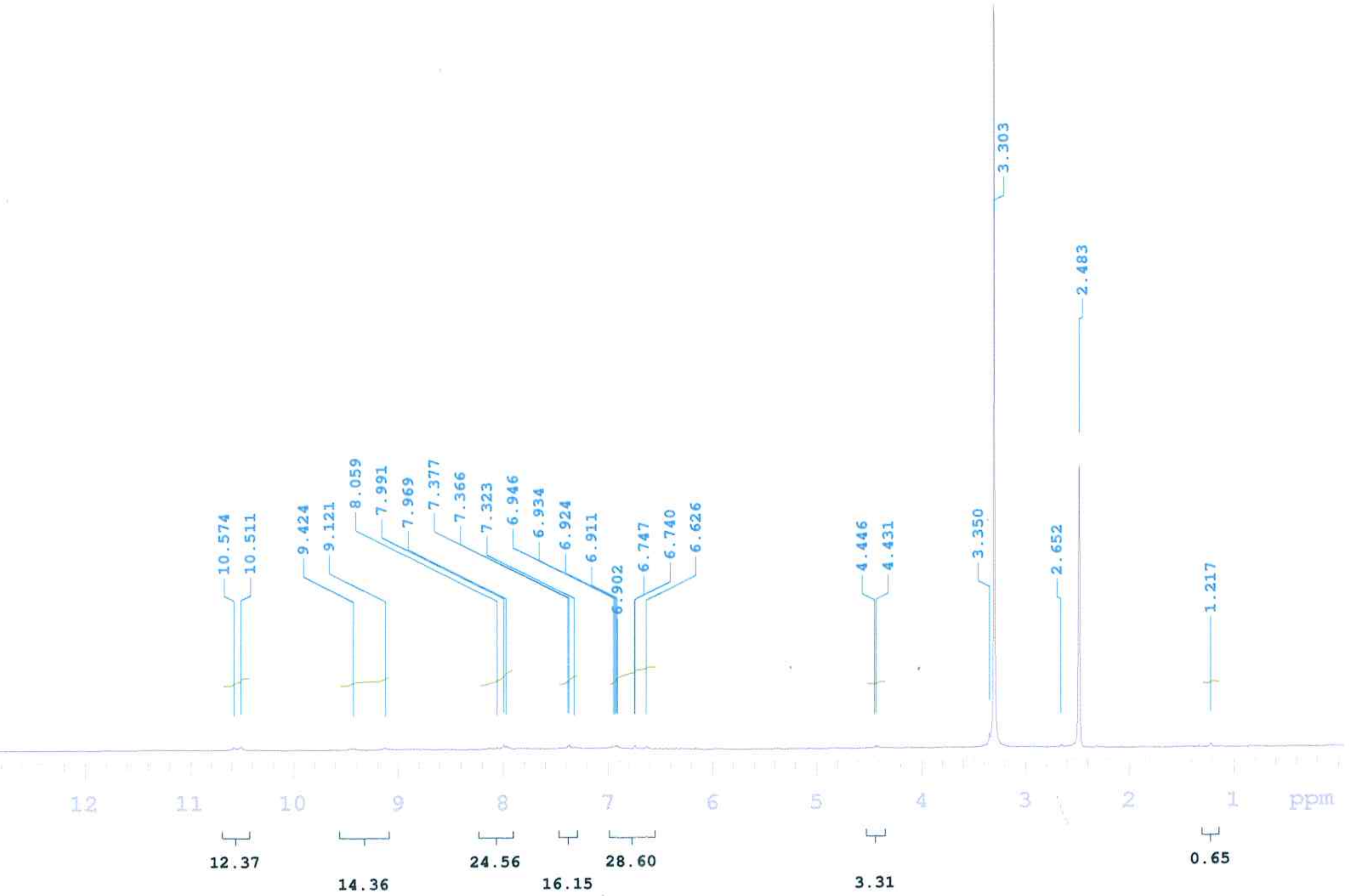

Data file /home/data/NMRlab2019/Feb/Dr-EmanHassan-E4_20190224_01/Dr-EmanHassan-E4_PROTON_01

Plot date 2019-03-09

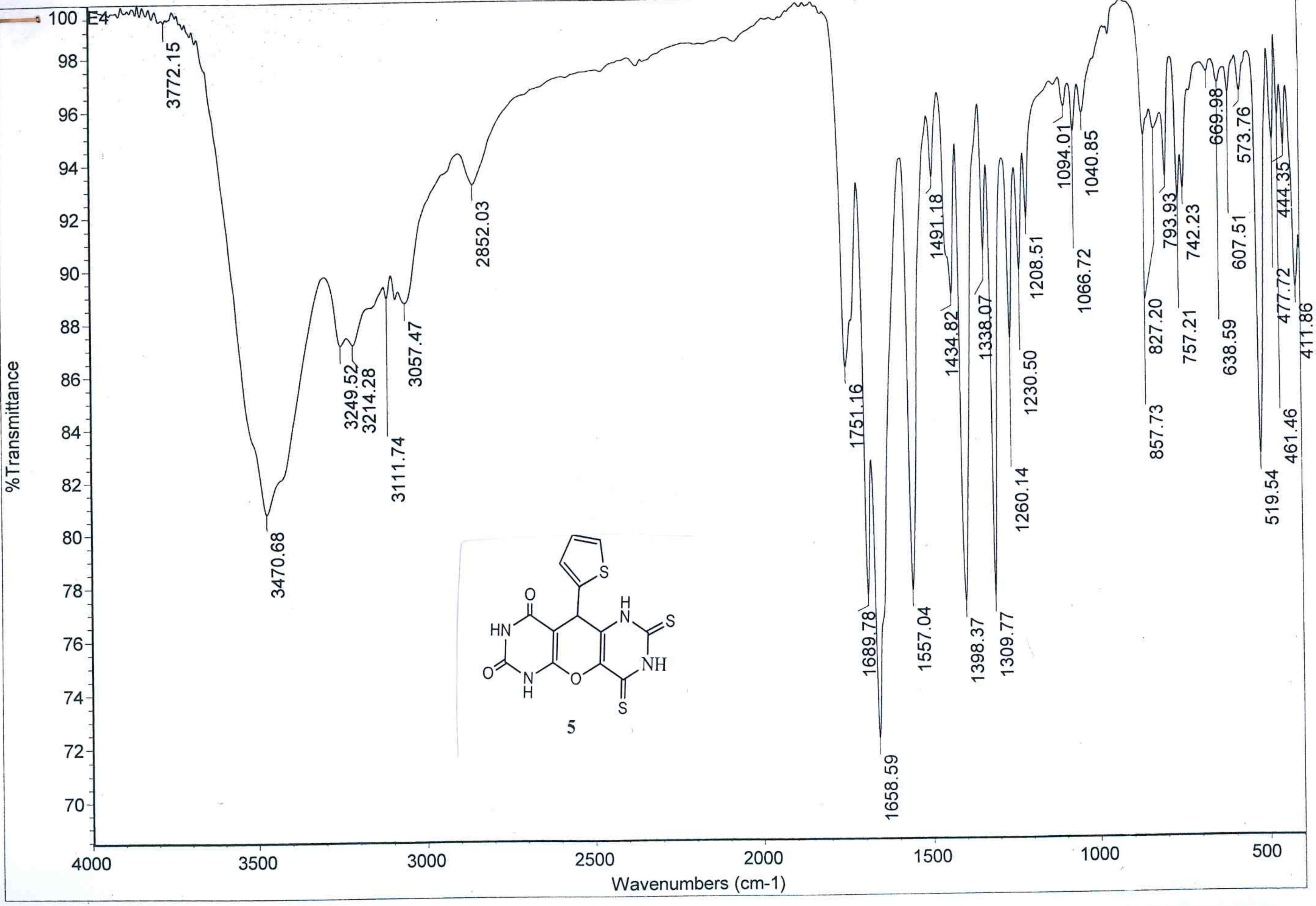
E4
%Transmittance
Wavenumbers (cm-1)
100
98
96
94
92
90
88
86
84
82
80
78
76
74
72
70
4000
3500
3000
2500
2000
1500
1000
500
3772.15
3470.68
3249.52
3214.28
3111.74
3057.47
2852.03
1751.16
1689.78
1658.59
1557.04
1491.18
1434.82
1398.37
1338.07
1309.77
1260.14
1230.50
1208.51
1094.01
1066.72
1040.85
857.73
827.20
793.93
757.21
742.23
669.98
638.59
607.51
573.76
519.54
477.72
461.46
444.35
411.86
5

Dr-EmanHassan-E5

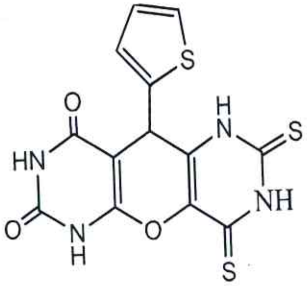

5

Dr-EmanHassan-E5

Sample Name	Dr-EmanHassan-E5	Pulse sequence	PROTON	Temperature	25	Laboratory	MODCL
Date collected	2019-02-24	Solvent	DMSO	Spectrometer	nmr400-mercury400	NMR User	

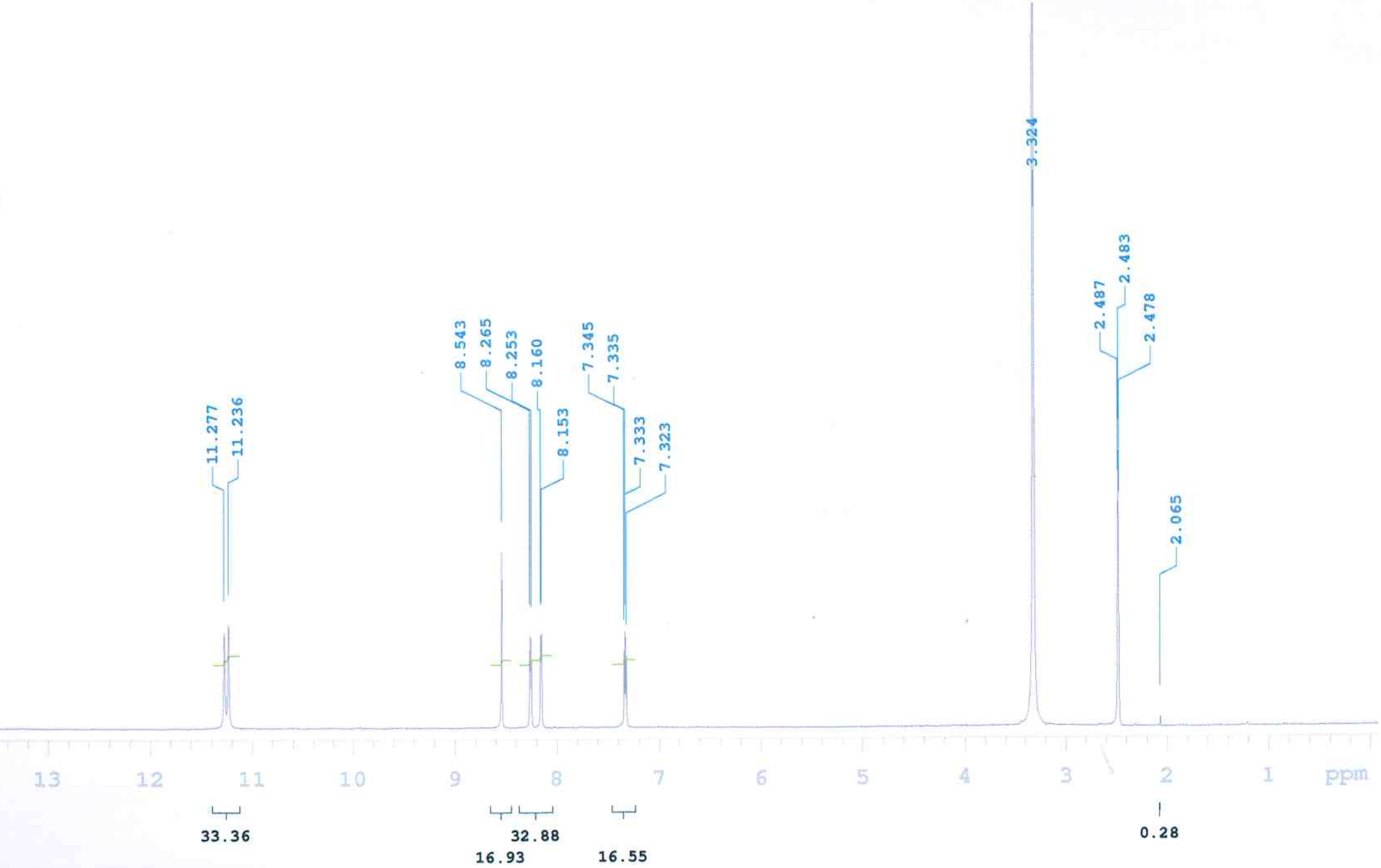

Data file /home/data/NMRlab2019/Feb/Dr-EmanHassan-E5_20190224_01/Dr-EmanHassan-E5_PROTON_01

Plot date 2019-03-09

Dr-EmanHassan-E5

Dr-EmanHassan-E5

Sample Name	Dr-EmanHassan-E5	Pulse sequence	CARBON	Temperature	25	Laboratory	MODCL
Date collected	2019-02-24	Solvent	DMSO	Spectrometer	nmr400-mercury400	NMR User	

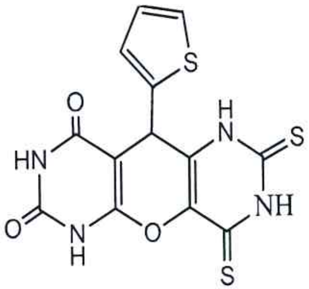

5

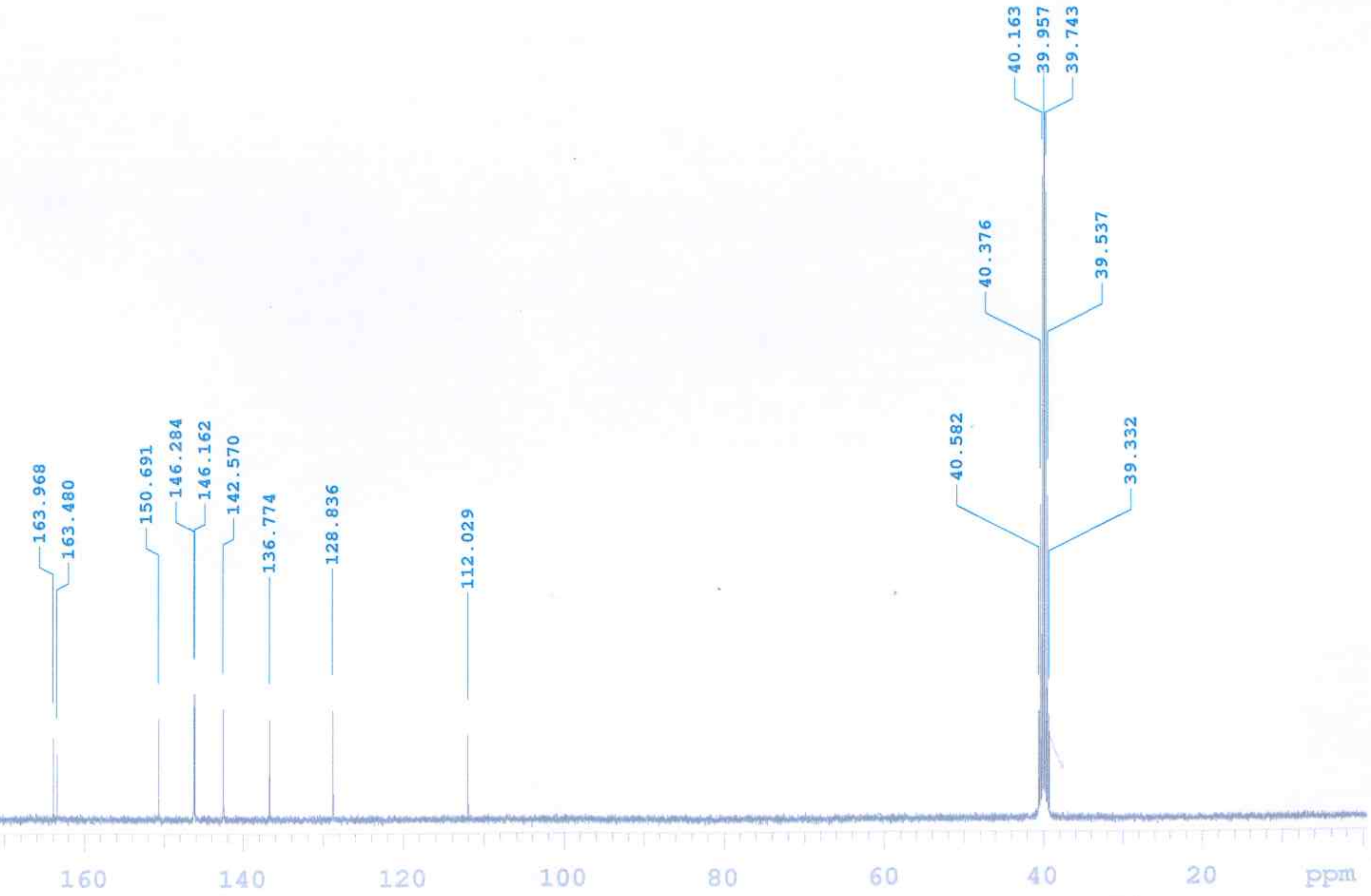

Data file /home/data/NMRlab2019/Feb/Dr-EmanHassan-E5_20190224_01/Dr-EmanHassan-E5_CARBON_01

Plot date 2019-03-09

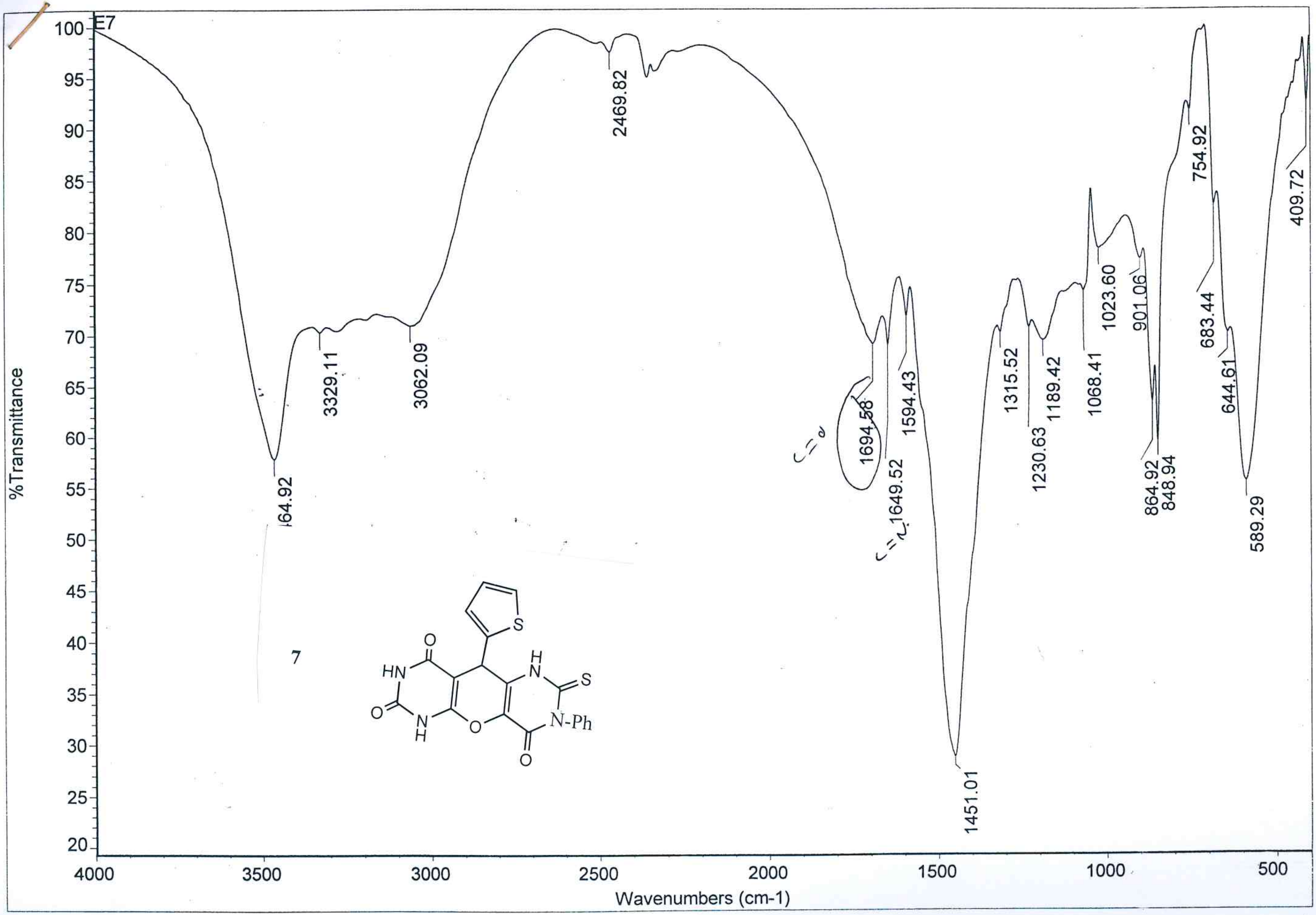

E7
%Transmittance
Wavenumbers (cm-1)
100
95
90
85
80
75
70
65
60
55
50
45
40
35
30
25
20
4000
3500
3000
2500
2000
1500
1000
500
164.92
3329.11
3062.09
2469.82
1694.58
1649.52
1594.43
1451.01
1315.52
1230.63
1189.42
1068.41
1023.60
901.06
864.92
848.94
754.92
683.44
644.61
589.29
409.72
7
HN
O
N
H
S
N-Ph

Dr-EmanHassan-E7

Dr-EmanHassan-E7

Sample Name	Dr-EmanHassan-E7	Pulse sequence	PROTON	Temperature	25	Laboratory	MODCL
Date collected	2019-02-24	Solvent	DMSO	Spectrometer	nmr400-mercury400	NMR User	Cap-Ahmed zaher

7

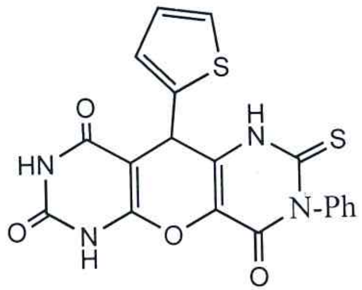

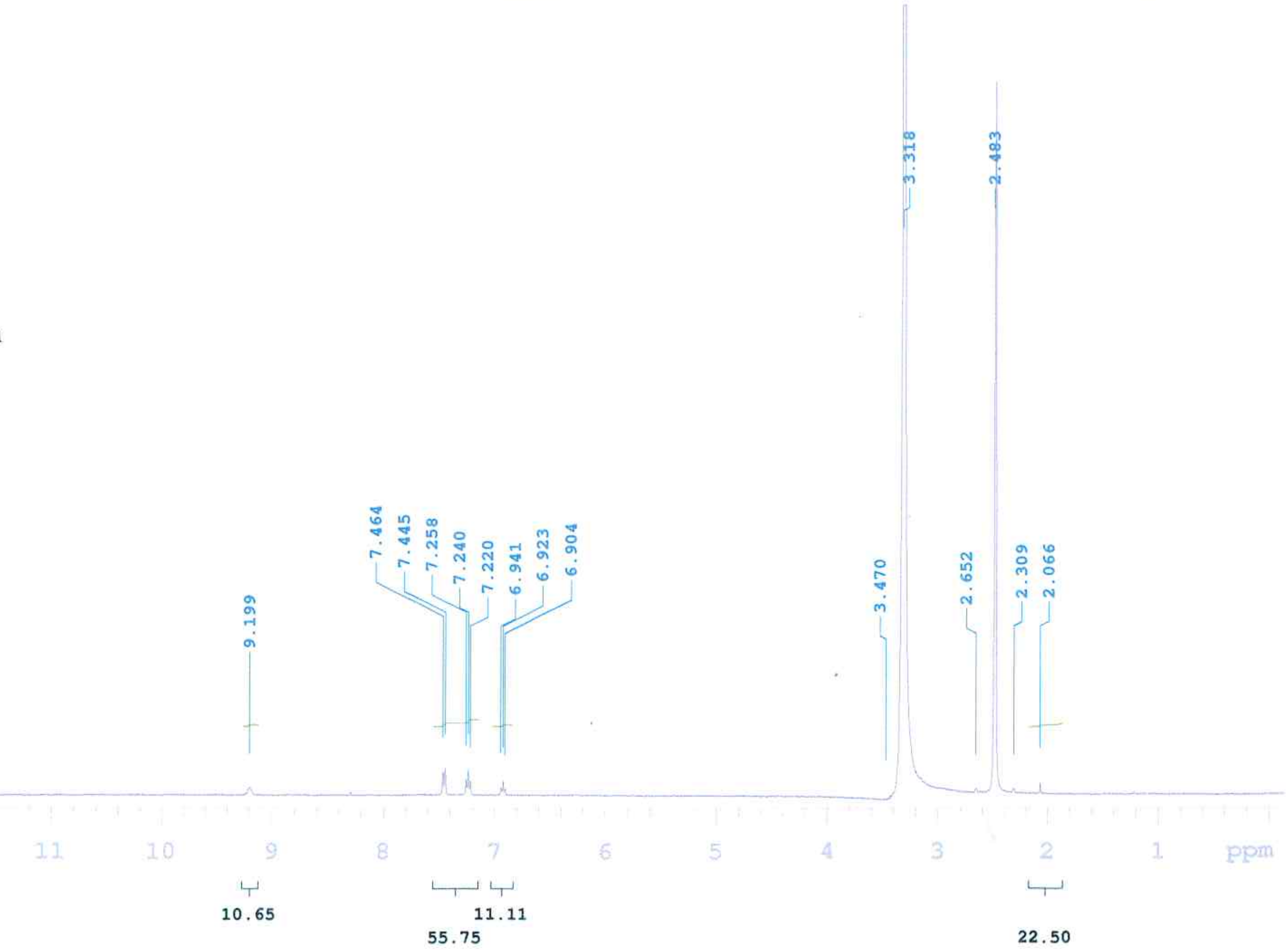

Data file /home/data/NMRlab2019/Feb/Dr-EmanHassan-E7_20190224_01/Dr-EmanHassan-E7_PROTON_01

Plot date 2019-03-09

Dr_EmanHassan-E6

Sample Name Dr_EmanHassan-E6	Pulse sequence PROTON	Temperature 25	Laboratory MODCL
Date collected 2018-09-16	Solvent DMSO	Spectrometer nmr400-mercury400	NMR User sameeh_Albadawy

Dr_EmanHassan-E6

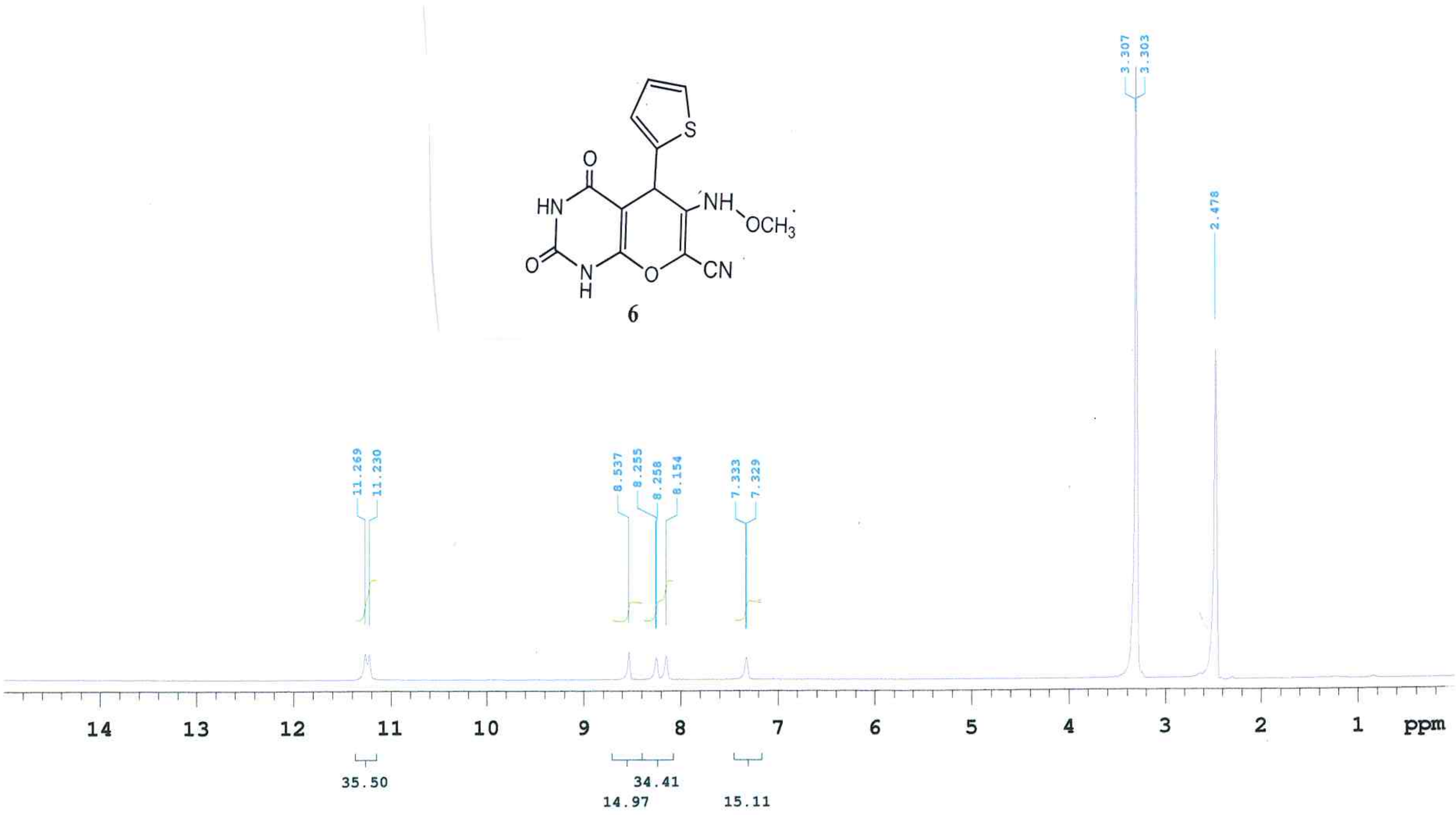

Plotname: Dr_EmanHassan-E6_PROTON_01_plot02

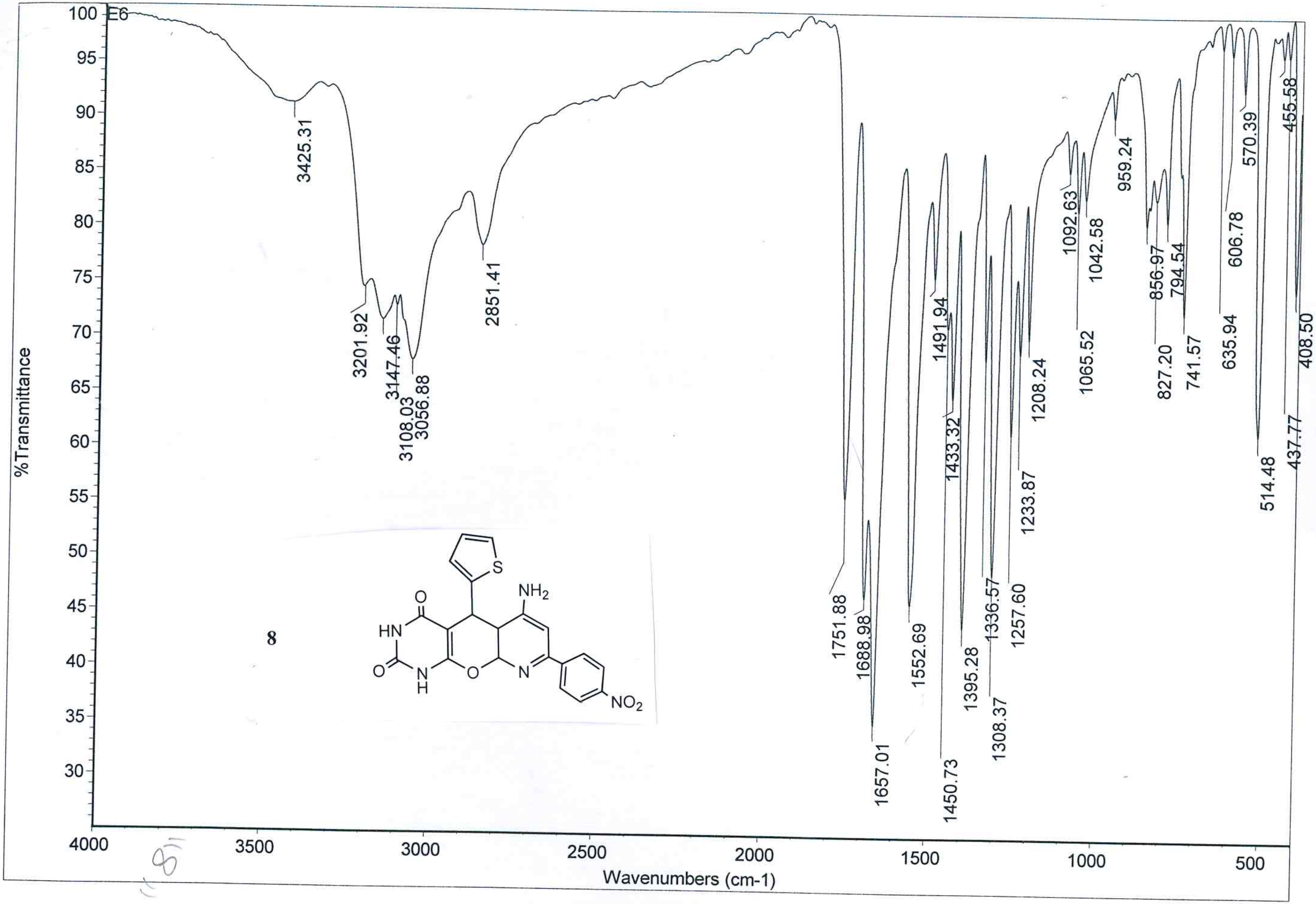
E6
%Transmittance
Wavenumbers (cm-1)
100
95
90
85
80
75
70
65
60
55
50
45
40
35
30
4000
3500
3000
2500
2000
1500
1000
500
3425.31
3201.92
3147.46
3108.03
3056.88
2851.41
1751.88
1688.98
1657.01
1552.69
1491.94
1450.73
1433.32
1395.28
1336.57
1308.37
1257.60
1233.87
1208.24
1092.63
1065.52
1042.58
959.24
856.97
827.20
794.54
741.57
635.94
606.78
570.39
514.48
455.58
437.77
408.50
8
O
HN
O
N
H
O
N
S
NH2
NO2

(8)

Dr-EmanHassan-E8

Dr-EmanHassan-E8

Sample Name	**Dr-EmanHassan-E8**	Pulse sequence	**PROTON**	Temperature	**25**	Laboratory	**MODCL**
Date collected	**2019-02-23**	Solvent	**DMSO**	Spectrometer	**nmr400-mercury400**	NMR User	

8

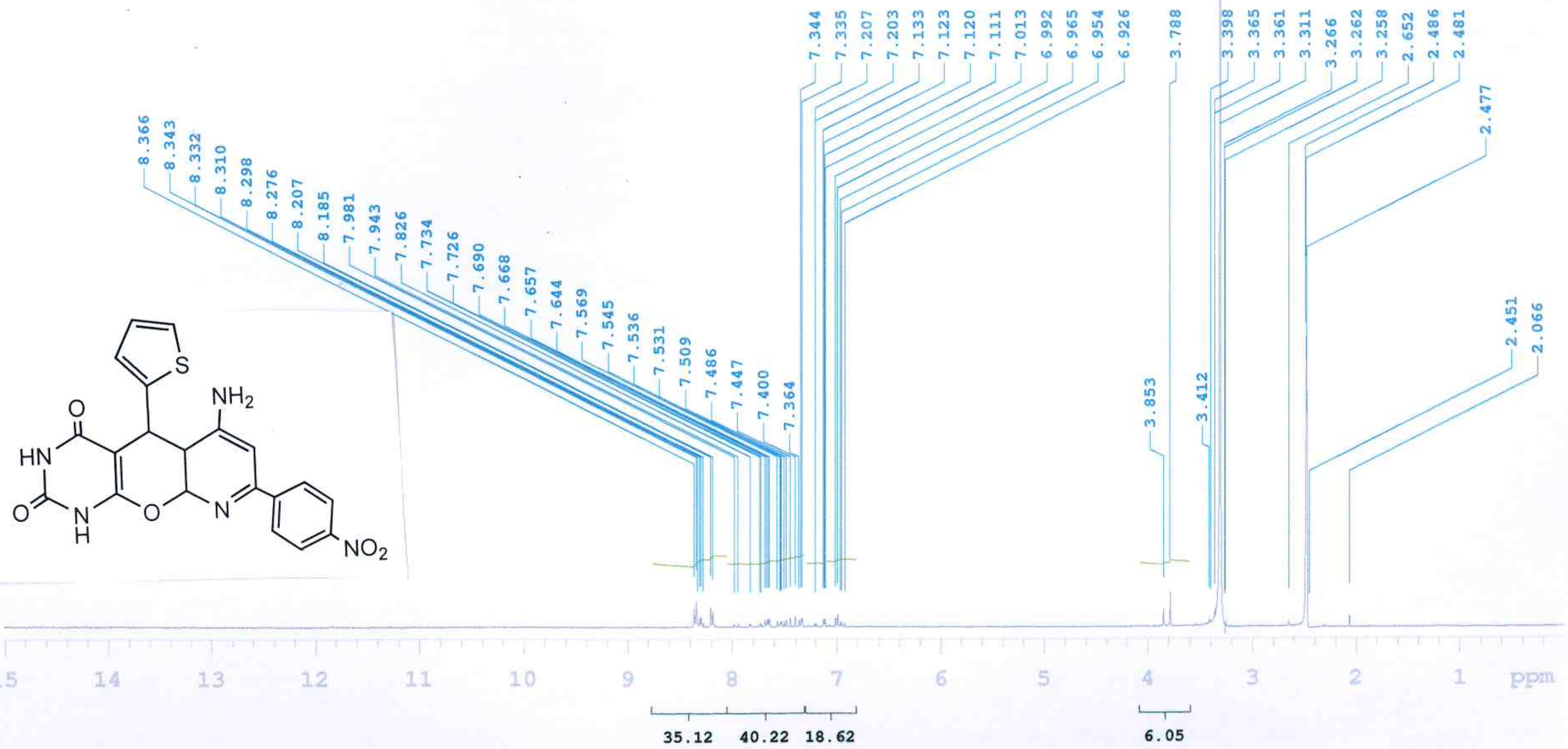

Data file /home/data/NMRlab2019/Feb/Dr-EmanHassan-E8_20190223_01/Dr-EmanHassan-E8_PROTON_01

Plot date 2019-03-09

Dr-EmanHassan-E8

Dr-EmanHassan-E8

Sample Name	**Dr-EmanHassan-E8**	Pulse sequence	**CARBON**	Temperature	**25**	Laboratory	**MODCL**
Date collected	**2019-02-23**	Solvent	**DMSO**	Spectrometer	**nmr400-mercury400**	NMR User	

8

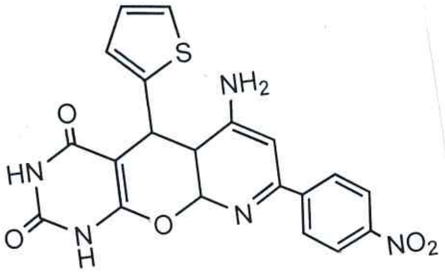

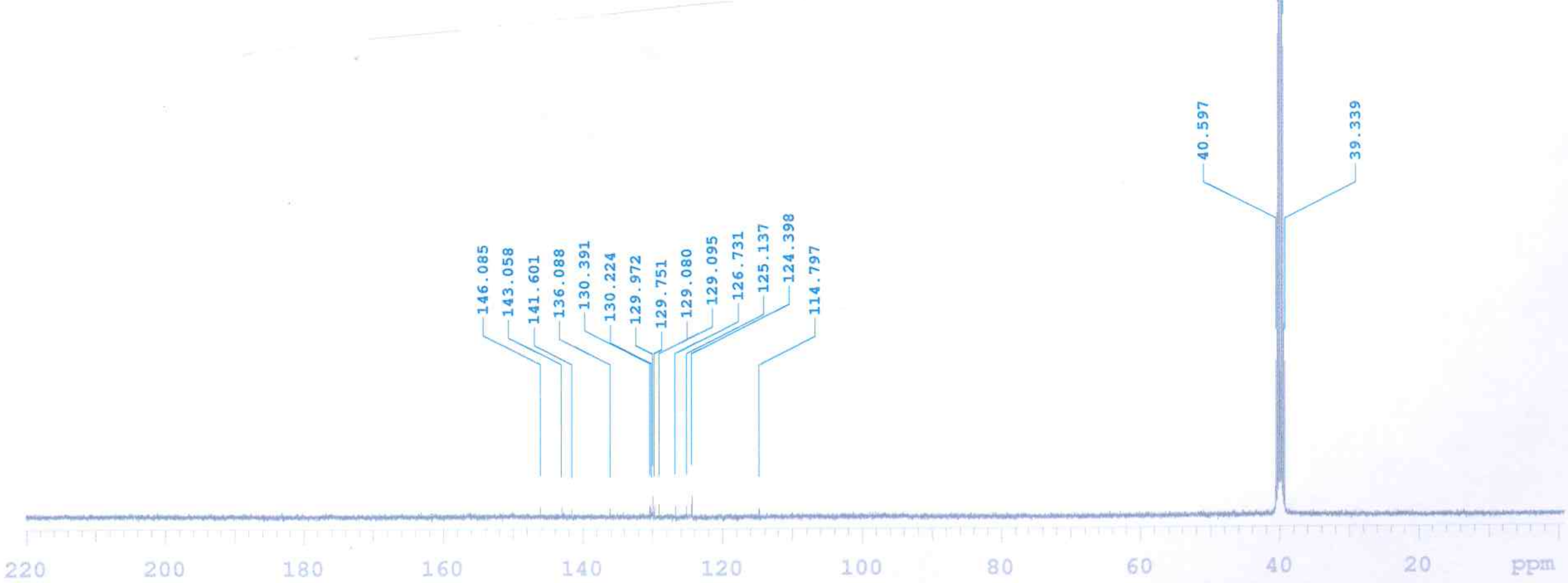

Data file /home/data/NMRlab2019/Feb/Dr-EmanHassan-E8_20190223_01/Dr-EmanHassan-E8_CARBON_01

Plot date 2019-03-09

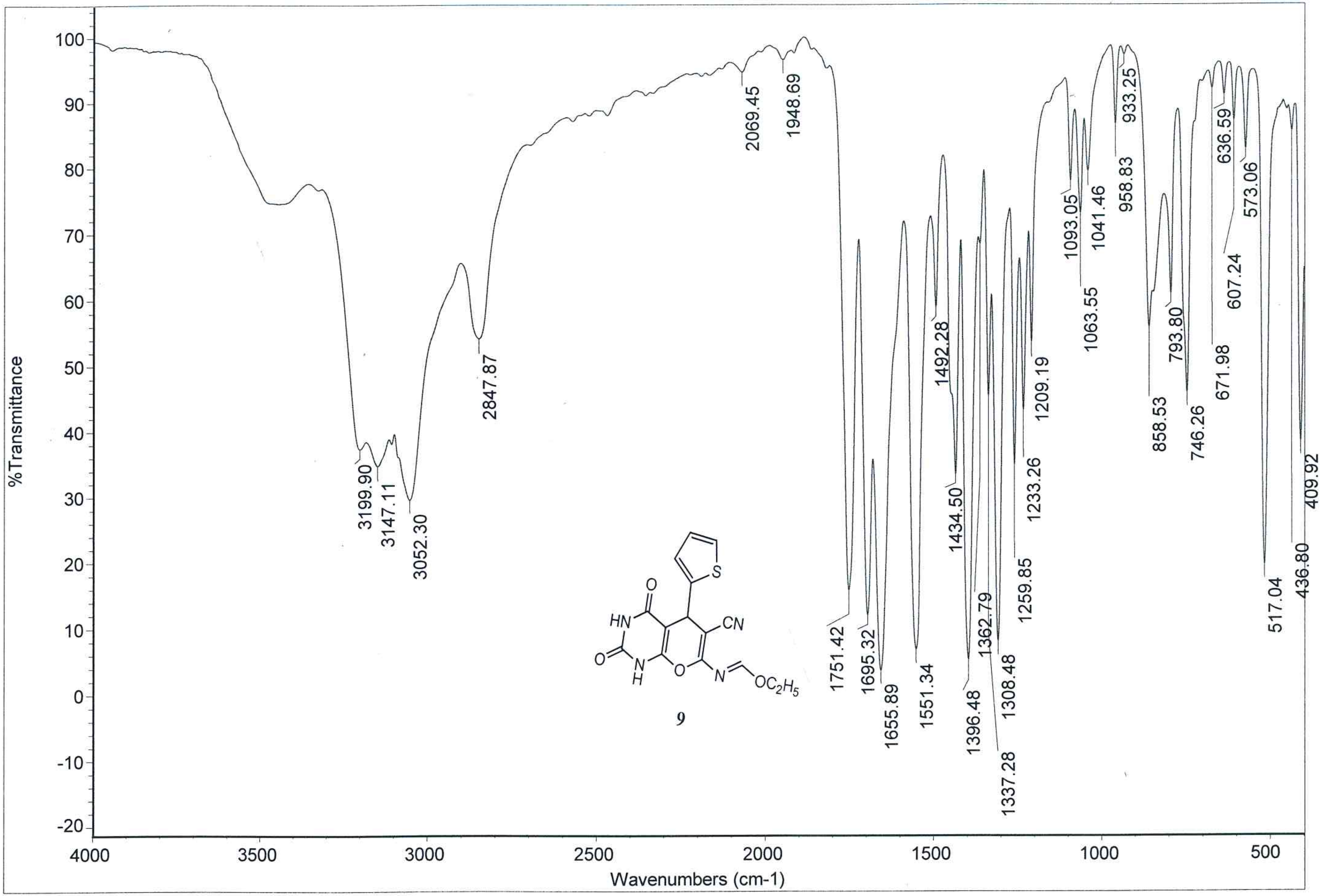
%Transmittance
Wavenumbers (cm-1)
100
90
80
70
60
50
40
30
20
10
0
-10
-20
4000
3500
3000
2500
2000
1500
1000
500
3199.90
3147.11
3052.30
2847.87
2069.45
1948.69
1751.42
1695.32
1655.89
1551.34
1492.28
1434.50
1396.48
1362.79
1337.28
1308.48
1259.85
1233.26
1209.19
1093.05
1063.55
1041.46
958.83
933.25
858.53
793.80
746.26
671.98
636.59
607.24
573.06
517.04
436.80
409.92
S
O
HN
CN
O
N
H
O
N
OC2H5
9

(6)

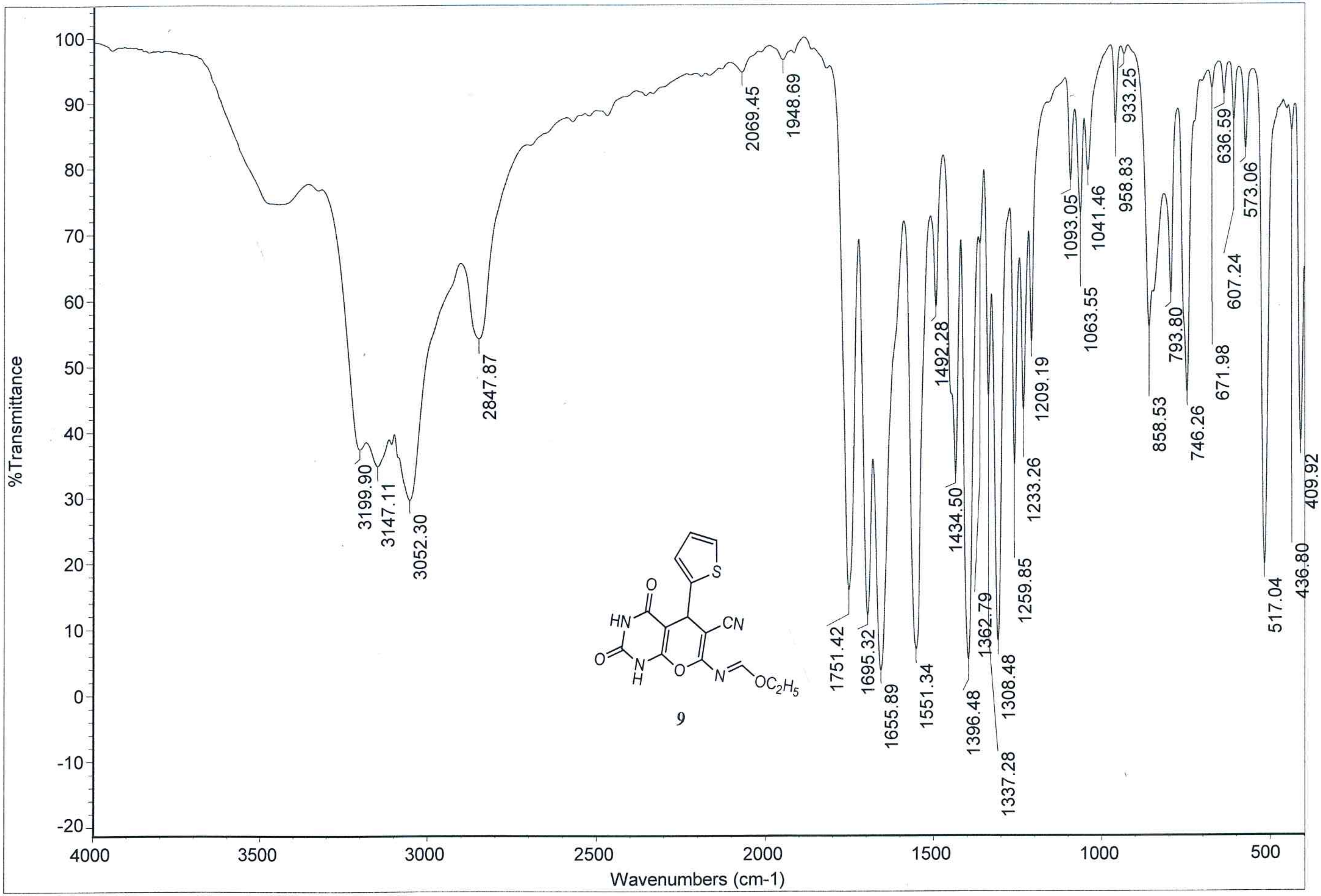
%Transmittance
Wavenumbers (cm-1)
100
90
80
70
60
50
40
30
20
10
0
-10
-20
4000
3500
3000
2500
2000
1500
1000
500
3199.90
3147.11
3052.30
2847.87
2069.45
1948.69
1751.42
1695.32
1655.89
1551.34
1492.28
1434.50
1396.48
1362.79
1337.28
1308.48
1259.85
1233.26
1209.19
1093.05
1063.55
1041.46
958.83
933.25
858.53
793.80
746.26
671.98
636.59
607.24
573.06
517.04
436.80
409.92
HN
O
O
N
H
O
S
CN
N
OC2H5
9

(6)

Dr-EmanHassan-E9

Sample Name	Dr-EmanHassan-E9	Pulse sequence	PROTON	Temperature	25	Laboratory	MODCL
Date collected	2019-02-23	Solvent	DMSO	Spectrometer	nmr400-mercury400	NMR User	

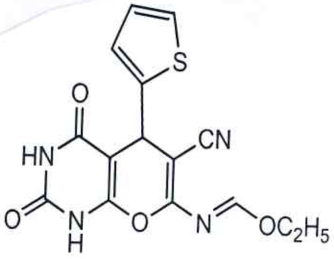

9

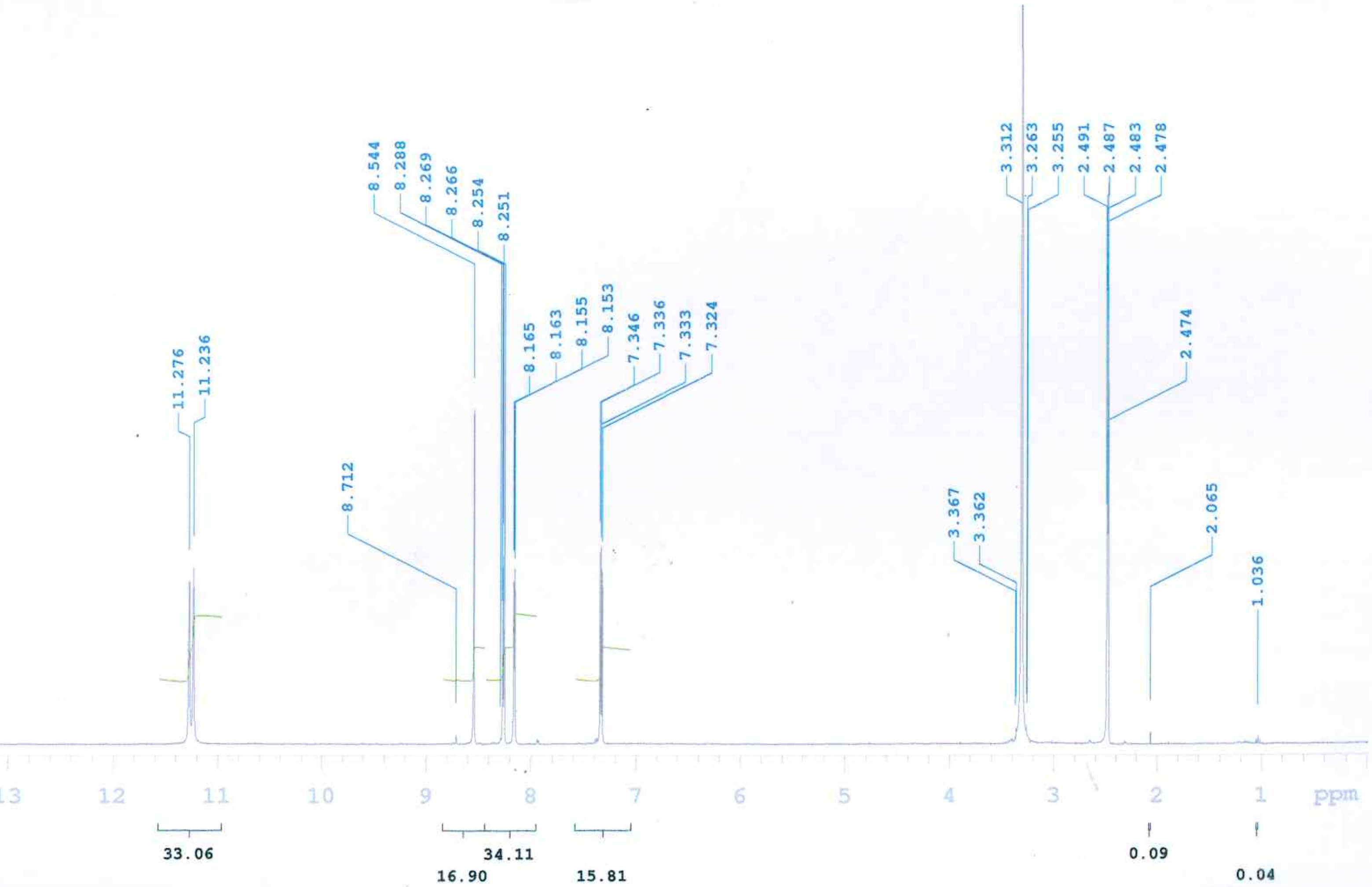

Data file /home/data/NMRlab2019/Feb/Dr-EmanHassan-E9_20190223_01.2/Dr-EmanHassan-E9_PROTON_01

Plot date 2019-03-09

Dr-EmanHassan-E9

Sample Name Dr-EmanHassan-E9	Pulse sequence CARBON	Temperature 25	Laboratory MODCL
Date collected 2019-02-23	Solvent DMSO	Spectrometer nmr400-mercury400	NMR User

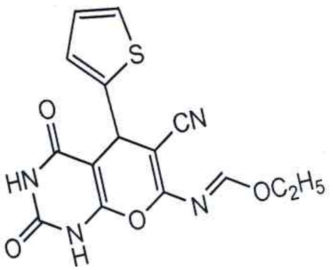

9

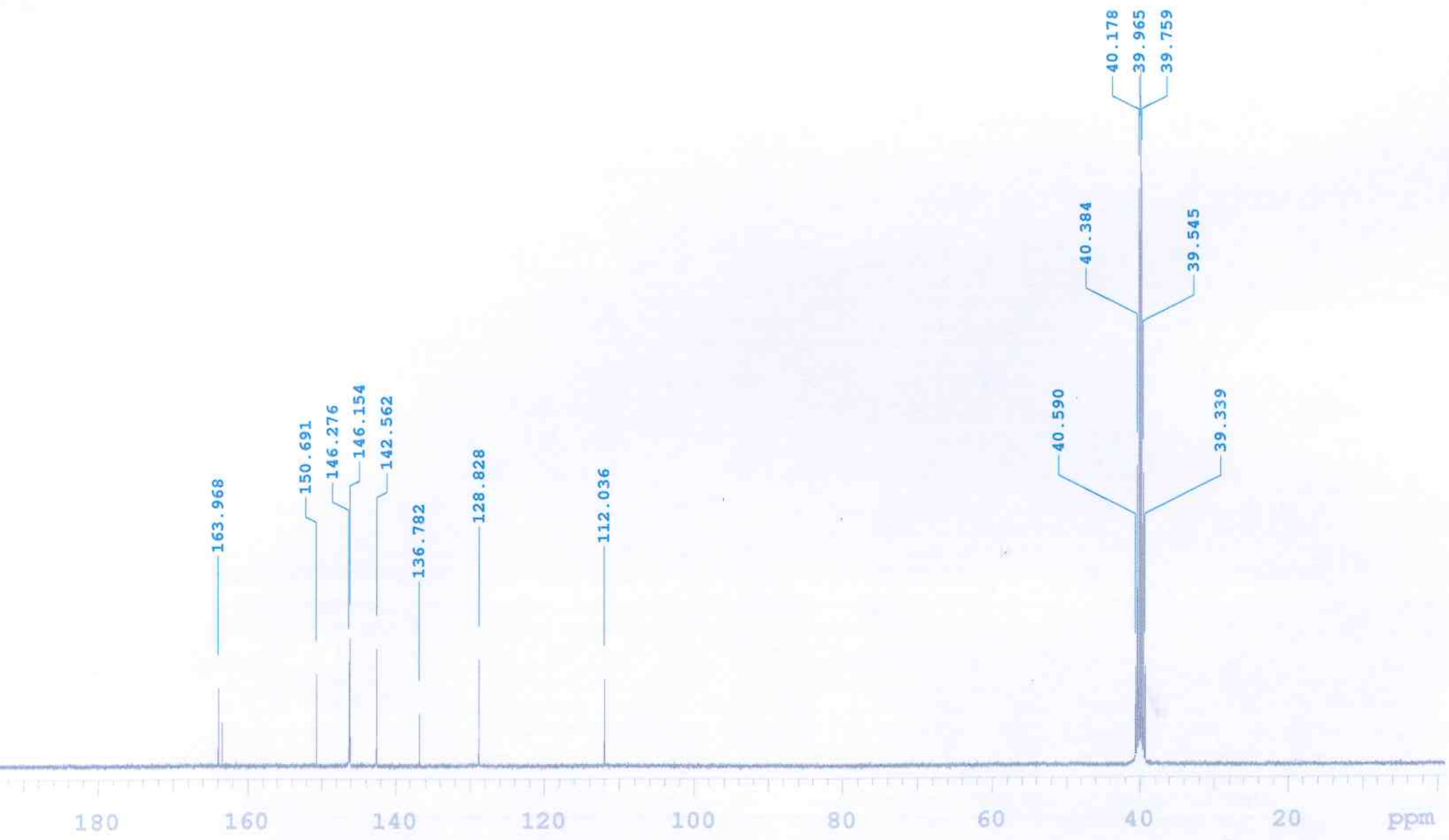

Data file /home/data/NMRlab2019/Feb/Dr-EmanHassan-E9_20190223_01/Dr-EmanHassan-E9_CARBON_01

Plot date 2019-03-09

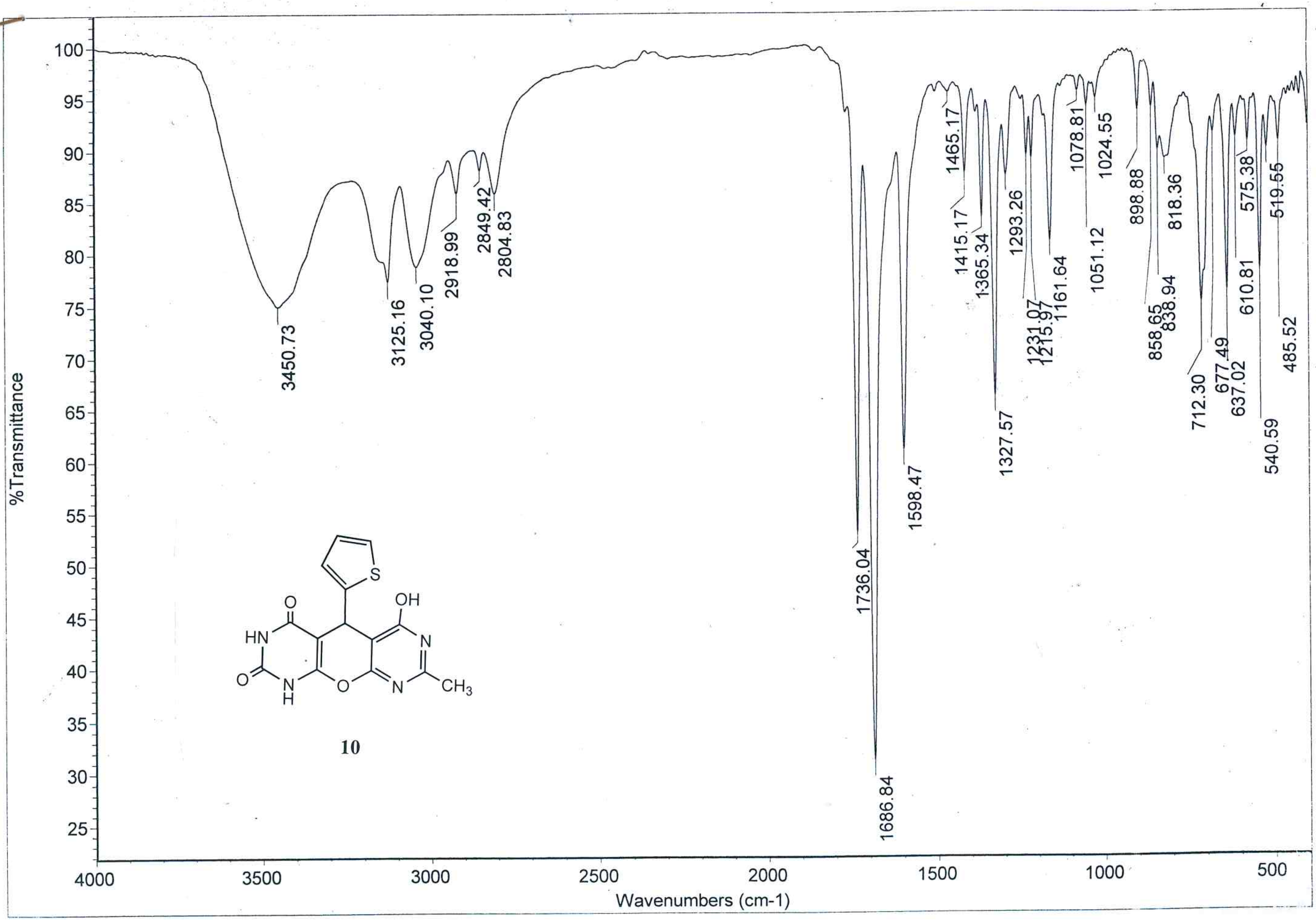

%Transmittance
Wavenumbers (cm-1)
100
95
90
85
80
75
70
65
60
55
50
45
40
35
30
25
4000
3500
3000
2500
2000
1500
1000
500
3450.73
3125.16
3040.10
2918.99
2849.42
2804.83
1736.04
1686.84
1598.47
1465.17
1415.17
1365.34
1327.57
1293.26
1231.07
1215.97
1161.64
1078.81
1051.12
1024.55
898.88
858.65
838.94
818.36
712.30
677.49
637.02
610.81
575.38
540.59
519.55
485.52
OH
O
S
HN
N
O
N
H
O
N
CH3
10

Dr_EmanHassan-10

Sample Name Dr_EmanHassan-10	Pulse sequence PROTON	Temperature 25	Laboratory MODCL
Date collected 2018-09-16	Solvent DMSO	Spectrometer nmr400-mercury400	NMR User sameeh_Albadawy

Dr_EmanHassan-10

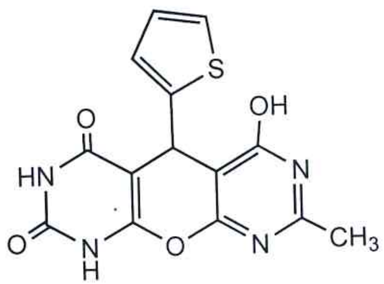

10

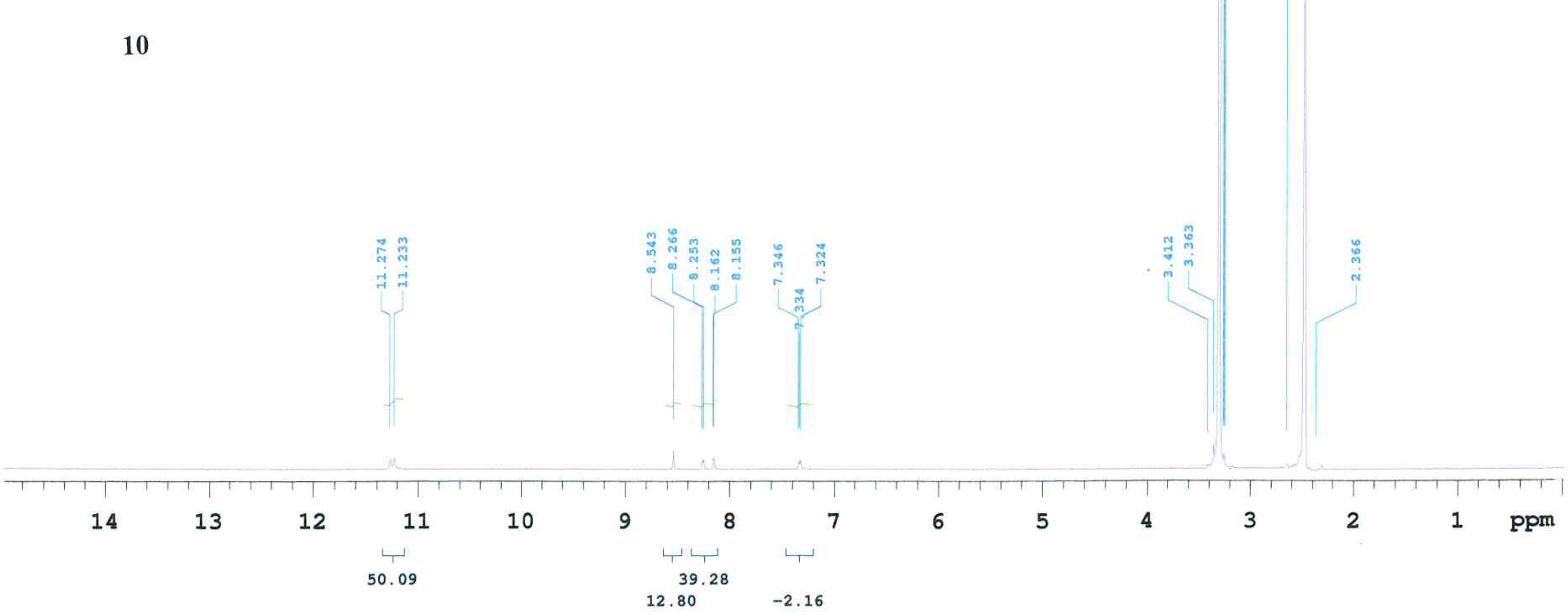

Plotname: Dr_EmanHassan-10_PROTON_01_plot02

Data file /home/data/NMRlab2018/Sep/Dr_EmanHassan-10_20180916_01/Dr_EmanHassan-10_PROTON_01

Plot date 2018-09-16

Dr-EmanHassan-E10

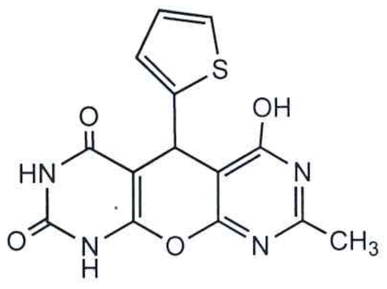

10

Dr-EmanHassan-E10

Sample Name	Dr-EmanHassan-E10	Pulse sequence	CARBON	Temperature	25	Laboratory	MODCL
Date collected	2019-02-24	Solvent	DMSO	Spectrometer	nmr400-mercury400	NMR User	

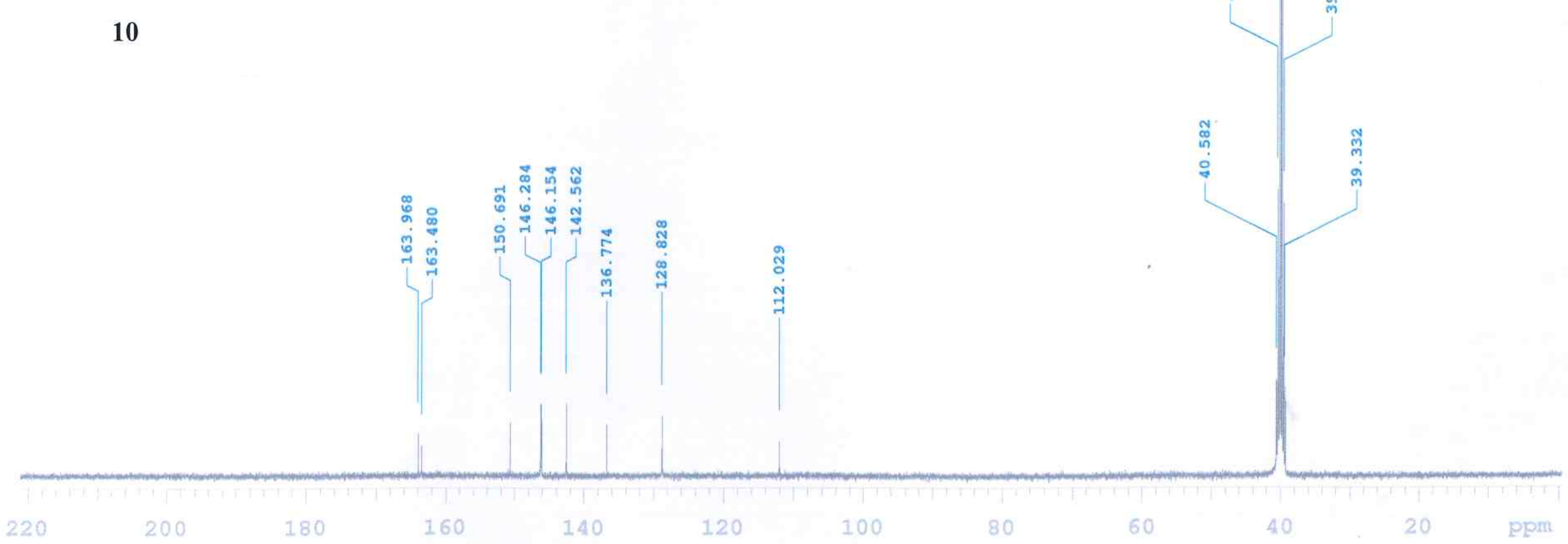

Data file /home/data/NMRlab2019/Feb/Dr-EmanHassan-E10_20190224_01/Dr-EmanHassan-E10_CARBON_01

Plot date 2019-03-09

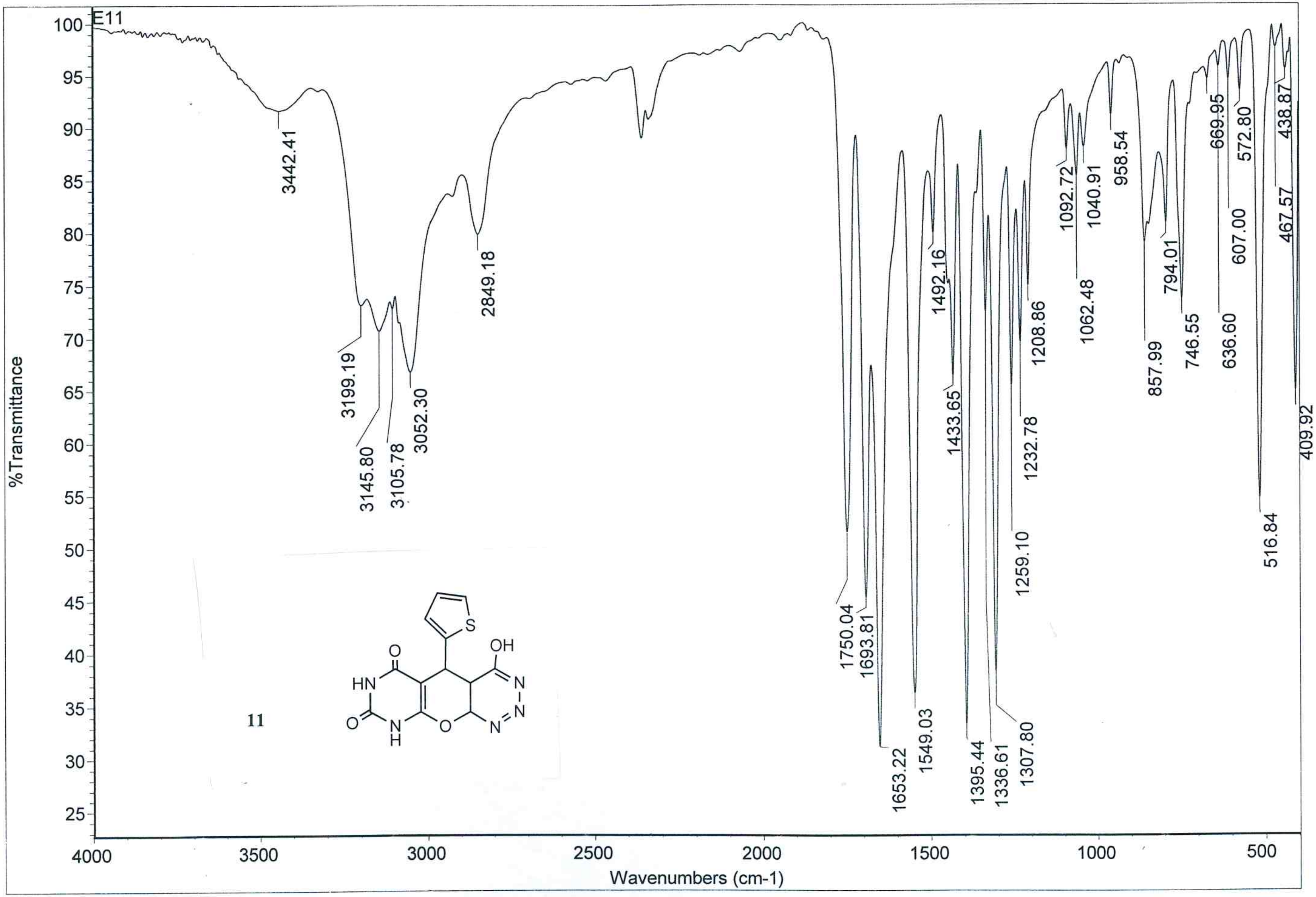
E11
%Transmittance
Wavenumbers (cm-1)
3442.41
3199.19
3145.80
3105.78
3052.30
2849.18
1750.04
1693.81
1653.22
1549.03
1492.16
1433.65
1395.44
1336.61
1307.80
1259.10
1232.78
1208.86
1092.72
1062.48
1040.91
958.54
857.99
794.01
746.55
669.95
636.60
607.00
572.80
516.84
467.57
438.87
409.92
S
OH
O
HN
N
N
O
N
H
O
N
11

Dr-EmanHassan-E11

Dr-EmanHassan-E11

Sample Name	Dr-EmanHassan-E11	Pulse sequence	PROTON	Temperature	25	Laboratory	MODCL
Date collected	2019-02-24	Solvent	DMSO	Spectrometer	nmr400-mercury400	NMR User	

11

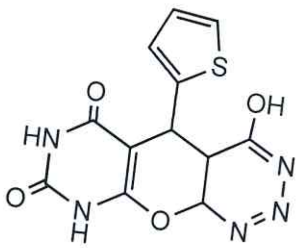

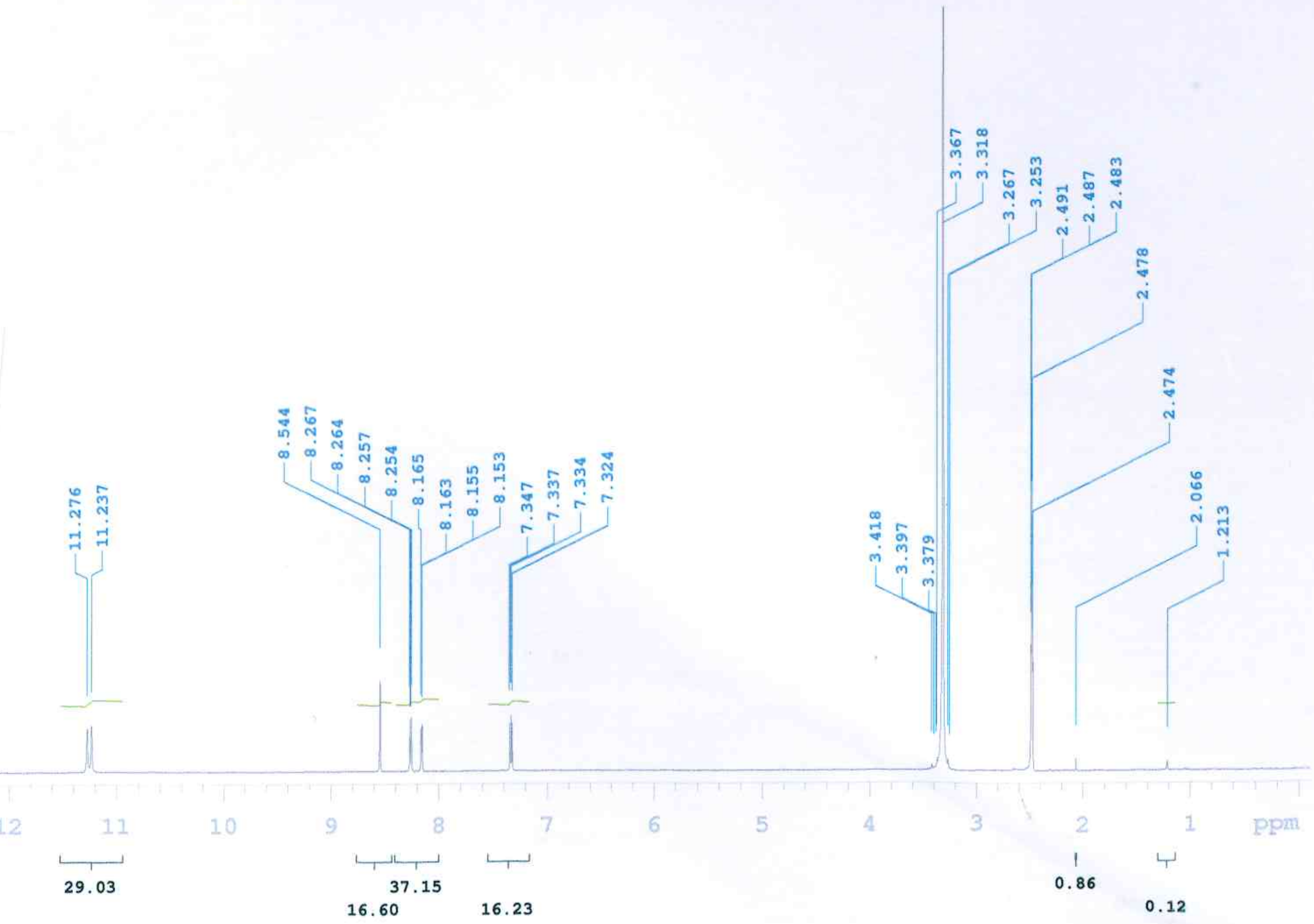

Data file /home/data/NMRlab2019/Feb/Dr-EmanHassan-E11_20190224_01/Dr-EmanHassan-E11_PROTON_01

Plot date 2019-03-09

Dr-EmanHassan-E11

Sample Name	Dr-EmanHassan-E11	Pulse sequence	CARBON	Temperature	25	Laboratory	MODCL
Date collected	2019-02-24	Solvent	DMSO	Spectrometer	nmr400-mercury400	NMR User	

Dr-EmanHassan-E11

11

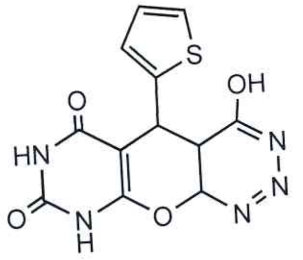

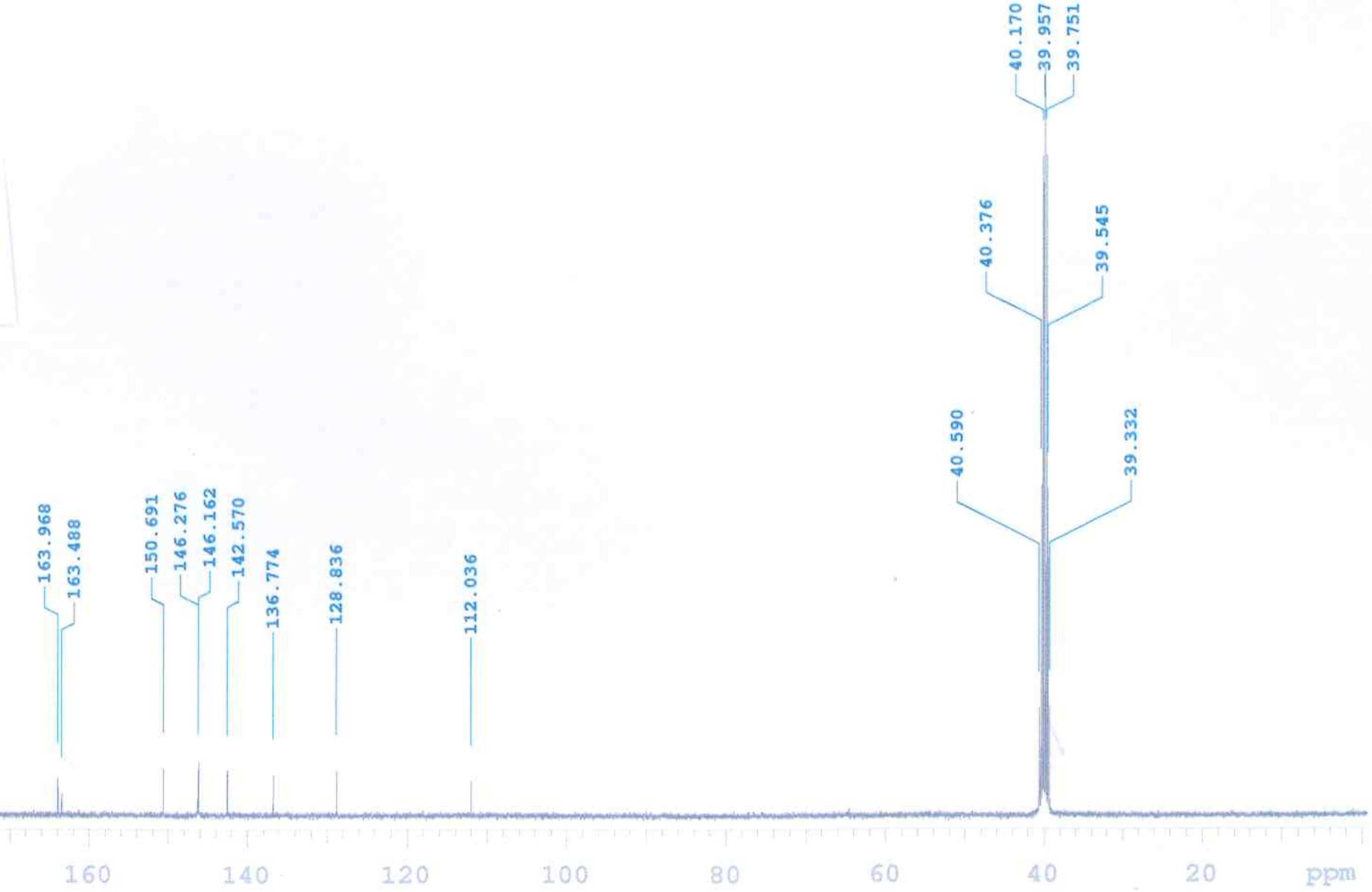

Data file /home/data/NMRlab2019/Feb/Dr-EmanHassan-E11_20190224_01/Dr-EmanHassan-E11_CARBON_01

Plot date 2019-03-09
